# A framework for modelling gene regulation which accommodates non-equilibrium mechanisms

**DOI:** 10.1186/s12915-014-0102-4

**Published:** 2014-12-05

**Authors:** Tobias Ahsendorf, Felix Wong, Roland Eils, Jeremy Gunawardena

**Affiliations:** DKFZ, Heidelberg, D-69120 Germany; Harvard College, Cambridge, 02138 USA; Department of Systems Biology, Harvard Medical School, 200 Longwood Avenue, Boston, 02115 USA; Institute of Pharmacy and Molecular Biotechnology (IPMB) and BioQuant, University of Heidelberg, Heidelberg, Germany

**Keywords:** linear framework, Laplacian dynamics, thermodynamic formalism, non-equilibrium statistical mechanics, gene regulation, chromatin domains, steroid-hormone responsive genes, phosphate regulation, *PHO5*, product graph, independence

## Abstract

**Background:**

Gene regulation has, for the most part, been quantitatively analysed by assuming that regulatory mechanisms operate at thermodynamic equilibrium. This formalism was originally developed to analyse the binding and unbinding of transcription factors from naked DNA in eubacteria. Although widely used, it has made it difficult to understand the role of energy-dissipating, epigenetic mechanisms, such as DNA methylation, nucleosome remodelling and post-translational modification of histones and co-regulators, which act together with transcription factors to regulate gene expression in eukaryotes.

**Results:**

Here, we introduce a graph-based framework that can accommodate non-equilibrium mechanisms. A gene-regulatory system is described as a graph, which specifies the DNA microstates (vertices), the transitions between microstates (edges) and the transition rates (edge labels). The graph yields a stochastic master equation for how microstate probabilities change over time. We show that this framework has broad scope by providing new insights into three very different *ad hoc* models, of steroid-hormone responsive genes, of inherently bounded chromatin domains and of the yeast *PHO5* gene. We find, moreover, surprising complexity in the regulation of *PHO5*, which has not yet been experimentally explored, and we show that this complexity is an inherent feature of being away from equilibrium. At equilibrium, microstate probabilities do not depend on how a microstate is reached but, away from equilibrium, each path to a microstate can contribute to its steady-state probability. Systems that are far from equilibrium thereby become dependent on history and the resulting complexity is a fundamental challenge. To begin addressing this, we introduce a graph-based concept of independence, which can be applied to sub-systems that are far from equilibrium, and prove that history-dependent complexity can be circumvented when sub-systems operate independently.

**Conclusions:**

As epigenomic data become increasingly available, we anticipate that gene function will come to be represented by graphs, as gene structure has been represented by sequences, and that the methods introduced here will provide a broader foundation for understanding how genes work.

**Electronic supplementary material:**

The online version of this article (doi:10.1186/s12915-014-0102-4) contains supplementary material, which is available to authorized users.

## Background

A quantitative approach to analysing gene regulation in terms of the interactions between transcription factors (TFs) and DNA was first developed for *λ* repressor in *Escherichia coli* [1]. In the eubacterial context, TFs bind and unbind from naked DNA and it was assumed that these processes quickly reach thermodynamic equilibrium. Equilibrium statistical mechanics could then be used to calculate the probability of DNA microstates, or patterns of TF binding to DNA. The gene-regulation function, which expresses the dependence of mRNA transcription rate on the concentrations of the TFs, was then calculated as an average over the microstate probabilities. This equilibrium “thermodynamic formalism” has been widely used to analyse gene regulation in eubacteria [2-6].

Eukaryotic genomes use several mechanisms that dissipate energy. These include epigenetic mechanisms, such as DNA methylation, nucleosome remodelling and post-translational modification and demodification of histones, transcription factors, transcriptional co-regulators and components of the transcriptional machinery, like RNA polymerase or Mediator. In each case, energy is expended to operate the mechanism, through consumption of intermediary metabolites such as ATP. Background metabolic processes maintain the concentration of such metabolites, thereby providing the free energy required away from thermodynamic equilibrium.

Despite the presence of such non-equilibrium mechanisms, the thermodynamic formalism has been widely used to analyse gene regulation in eukaryotes, including yeast [7], flies [8-13] and human cells [14], and has been extensively reviewed [15-19]. In most cases, non-equilibrium mechanisms have not been incorporated in these models. An exception has been work on nucleosome positioning [18], for which the argument was made that energy dissipation is used primarily to overcome energy barriers, after which nucleosomes and transcription factors reach equilibrium in competing for DNA, thereby allowing treatment within the thermodynamic formalism. While initially successful, more recent experimental work suggests that this does not fully explain nucleosome positioning and that it is important to take energy dissipation into account [20,21]. Several other recent studies have also begun to raise doubts about the validity of the equilibrium assumption [22-24].

The biological significance of energy dissipation is broadly understood; it is essential for life. Its deeper implications for the molecular context were first clarified by John Hopfield in a seminal study [25]. He showed that if a molecular mechanism operated at equilibrium, then there was an absolute upper bound to how well it could carry out certain information-processing tasks, such as achieving fidelity in mRNA or protein production. The source of this upper bound was the property of detailed balance (discussed below), which is a fundamental physical constraint on equilibrium systems. To get beyond this upper bound, it is essential to expend energy and to drive the system away from equilibrium so that detailed balance no longer holds. Hopfield put forward a kinetic proofreading scheme, which he showed could achieve unlimited error correction by expending sufficient energy. Subsequent work has refined this scheme [26,27] but the limitation in the capabilities of equilibrium mechanisms has been a fundamental insight.

Despite this understanding, the significance of non-equilibrium mechanisms in gene regulation remains unclear. Energy must evidently be expended to pack DNA into the nucleus and to organise chromatin mechanically but it seems unlikely that evolution would not also take advantage of energy dissipation for cellular information processing. From a different perspective, increasing amounts of epigenomic data are becoming available through high-throughput experimental projects [28-30]. Without being able to analyse rigorously the non-equilibrium mechanisms that give rise to such data, it seems unlikely that we will fully understand the epigenomic capabilities of eukaryotic DNA, whose role in both development and evolution is of considerable interest [31-33].

One of the barriers to progress here has been the absence of a mathematical framework that can accommodate non-equilibrium mechanisms in gene regulation. We have developed a graph-based, “linear framework” for timescale separation in biochemical systems [34-38], which is not limited to thermodynamic equilibrium. We show here how this can be adapted to the non-equilibrium mechanisms that are found in gene regulation. The framework yields a stochastic master equation for the probabilities of DNA microstates. An important feature of this equation is that it is linear (hence, “linear framework”). The non-linearities that are always present in biochemical systems are accommodated through labels on the edges of the graph, without the need for any approximation. If a system is at equilibrium, the linear framework reduces to the thermodynamic formalism. The framework offers a chemist’s perspective in terms of reactions and rates in place of a physicist’s perspective in terms of states and free energies, and exploits graph theory to calculate the steady-state probabilities of microstates.

The catalytic production of mRNA by RNA polymerase is fundamentally irreversible and dissipative. In the thermodynamic formalism, the rate of mRNA expression is treated as an average over the equilibrium states. With the framework introduced here, the dissipative steps taken by mRNA polymerase can be explicitly included in the model, when required. What is not addressed here are the dynamics of mRNAs and proteins and the resulting important issue of gene expression noise [39,40]. This has only recently been analysed in the context of gene regulatory architecture [41,42]. It is possible to accommodate the numbers of mRNA and protein molecules within a graph-based framework but this requires infinite graphs in contrast to the finite graphs used here. The question of whether the graph-theoretic methods introduced here can be extended to infinite graphs is very interesting but lies outside the scope of the present paper.

We have three broad aims here. First, we want to introduce the new framework and show that it can be broadly applied to different types of problems in gene regulation and chromatin organisation. We use it to analyse systematically three very different *ad hoc* models: of steroid-hormone responsive genes where detailed balance is still assumed, of inherently bounded chromatin domains where dissipation is critical but no specific gene is being regulated and of regulation of the yeast *PHO5* gene where non-equilibrium nucleosome remodelling is explicitly included and detailed balance cannot be assumed. Second, we show that the gene-regulation function of *PHO5* is surprisingly complex. We are able to explain this complexity as an inherent feature of non-equilibrium systems, which arises from the dependence on history away from equilibrium. The scope of this complexity appears not to have been experimentally explored and may reflect information-processing capabilities that could not be achieved at equilibrium. Our third aim is to begin the study of graphs that exhibit reduced complexity. We formulate a graph-theoretic concept of independence for non-equilibrium systems and show that history-dependent complexity collapses when systems operate independently of each other.

To make this paper broadly accessible, we begin with a non-technical description of the framework, introducing some key concepts and explaining how graph structures provide useful qualitative insights. We then explain how graphs are constructed in terms of specific biochemical processes acting on DNA and chromatin. The quantitative calculation of steady-state probabilities relies on previous work, which is brought together in the next section to make the paper as self-contained as possible. The remaining sections work through the results described above.

## Results

### A graph-theoretic view of gene regulation

We offer in this section a non-technical account of the linear framework as applied to gene regulation. The technical details are provided, along with references, in the section on ‘Calculating microstate probabilities at steady state’.

**Figure 1:**
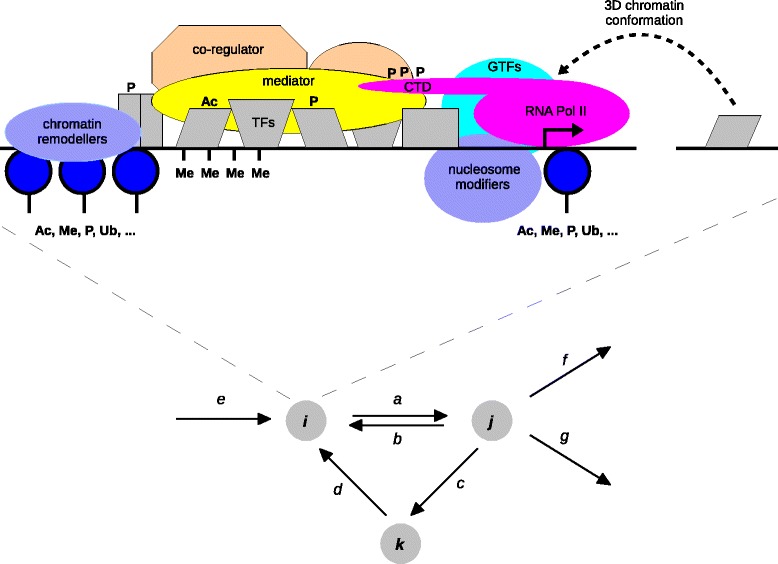
**Microstates and graphs.** A fragment of a graph is shown (below), with three vertices, *i*, *j* and *k*, and several labelled, directed edges. Vertex *i* is expanded into a microstate, or snapshot of a DNA state (above), showing some of the features that can be represented (not to scale). Here, a hypothetical promoter region of a gene is shown. Features include sequence-specific transcription factors bound to DNA (grey shapes), additional recruited components, such as transcriptional co-regulators (orange shapes), general-purpose transcriptional machinery, such as Mediator (yellow), general transcription factors (GTFs, blue-green) and RNA Pol II (magenta), along with chromatin remodellers and enzymatic factors that modify the histone tails of nucleosomes (blue shapes). Potential post-translational modifications of transcription factors, co-regulators and histone tails are shown by the corresponding symbols, along with DNA methylation. Distal enhancers may participate through 3D chromatin conformation, such as DNA looping. CTD is the carboxy terminal domain of RNA Pol II. 3D, three dimensional; CTD, carboxy terminal domain; GTF, general transcription factor; Pol, polymerase; Ac, acetylation; Me, methylation; P, phosphorylation; Ub, ubiquitination.

The framework starts with a labelled, directed graph consisting of a collection of vertices with directed edges between pairs of vertices and labels on the edges (Figure 1, bottom). The graphs considered here have only finitely many vertices and the edges always go between distinct vertices, so that there are no self-loops. It is further assumed that each graph is connected, which means that, given any two vertices, there is always a path of edges between them, ignoring edge directions. A connected graph is not in disjoint pieces.

The vertices of the graph correspond to microstates, or snapshots of DNA and its accompanying proteins. Figure 1 (top) shows the range of features that can potentially be found in a microstate, including TFs, transcriptional co-regulators, RNA polymerase, nucleosomes, chromatin remodelling enzymes, DNA looping, various forms of post-translational modification and DNA methylation. The directed edges correspond to transitions between microstates arising from biochemical reactions taking place on chromatin, such as the binding and unbinding of TFs or co-regulators or post-translational modification or demodification of proteins bound to DNA. Directed graphs of this kind are often found in the literature as qualitative summaries of the behaviour of regulatory mechanisms. Such cartoons can be given a rigorous mathematical basis through the methods introduced here.

The labels on the edges supply quantitative information in the form of effective rate constants for the corresponding transitions. Each label has units of inverse time, as in per second. The rate of some transitions, such as binding events, can depend on the concentration of components in solution around DNA. The labels can therefore be compound expressions involving component concentrations as well as kinetic parameters. In this way biochemical non-linearity is accommodated in the labels. An important feature of the framework is that the numerical values of the parameters do not have to be known in advance. They can be treated as symbols and many properties of the system can be calculated in symbolic form. This permits analysis without having to measure or estimate the actual values of the parameters.

The level of granularity used for the microstates, and the corresponding transitions, is a matter of choice. It can range from coarse-grained descriptions of open and closed chromatin to fine-grained descriptions of DNA sequence, individual nucleosomes and specific histone modifications. The choice depends on the context, the available experimental methods and data and the biological questions being asked. The graph constitutes a mathematical model of the system being studied and is best thought of not as a description of reality but as a precise statement of the assumptions being made about that reality – a hypothesis – from which rigorous deductions can be made and experiments proposed [43].

Because there is only one molecule of DNA, the dynamical behaviour of microstates has to be understood in terms of probabilities. If we imagine watching DNA over time, the microstates will fluctuate as transitions take place due to random molecular events, such as binding or unbinding of components. Let us denote the probability of the system being in microstate *i* at time *t* by *u*_*i*_(*t*). The following thought experiment may help to interpret this quantity. Imagine a large number of copies of the system being created in the identical starting condition at time 0, with the same initial microstate and the same protein components present in the surrounding solution at the same concentrations. As time progresses, the randomness of molecular events will cause the different copies of the system to diverge so that different microstates will be found in each system copy. The proportion of copies in which microstate *i* is found at time *t* is an approximation for *u*_*i*_(*t*) and this approximation becomes more accurate as the number of copies is increased. In other words, *u*_*i*_(*t*) measures how often microstate *i* will be found at time *t*, were it possible to repeatedly replay the system from its initial condition at time 0.

Probabilities can appear difficult to reason with but the graph-based framework offers a different way to think about them which may be more familiar. The vertices of the graph are regarded as chemical species with concentrations, the edges as chemical reactions and the labels as rate constants. Each reaction has only a single substrate and only a single product, like an isomerisation, so the graph describes a kind of one-dimensional chemistry. This macroscopic interpretation allows us to reason about concentrations and reactions but gives the same results as the microscopic interpretation in terms of probabilities and transitions. In other words, if we imagine placing concentrations of matter at each vertex and allowing the chemistry to work, then the change in concentrations over time is identical to the change in probabilities over time. The only thing we have to remember is that probabilities add up to 1 – the system must be in some microstate – so that the total concentration of matter at all vertices should be kept at 1. Because the reactions only move matter between vertices, and neither create nor destroy it, the total concentration remains the same over time (see Equation 2 below), so we only need to make it 1 to begin with.

**Figure 2:**
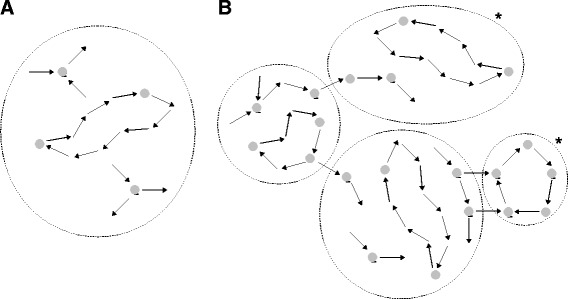
**Strongly connected graphs and components.** Outlines of hypothetical graphs are shown, omitting some vertices and edges and all labels. **(A)** A strongly connected graph in which any pair of vertices can be joined, both ways, by a path of contiguous edges in the same direction (central motif). **(B)** A graph that is not strongly connected can always be decomposed into maximal strongly connected sub-graphs, called strongly connected components (SCCs). The graph shown here has four SCCs demarcated by the dotted lines. In the macroscopic interpretation of one-dimensional chemistry, matter can only flow in one direction between SCCs, so that it eventually accumulates only on the terminal SCCs (marked with an asterisk). In the microscopic interpretation, microstates that are not in a terminal SCC have zero steady-state probability.

It is easy to imagine that, no matter what initial concentrations of matter are distributed over the vertices, the one-dimensional chemistry will eventually reach a steady state, in which production and consumption of each species are in balance and the concentration of each species is unchanging. Such a steady state occurs no matter what the structure of the graph. In a general graph, the steady state can depend on the initial concentrations that were chosen at time 0, so that there is a memory of these initial conditions (see the section ‘Formation of an inherently bounded chromatin domain’). However, if the graph is strongly connected, such memory is lost and the steady state becomes independent of the initial conditions and depends only on the structure of the graph. A strongly connected graph is one in which any pair of vertices are connected, both ways, by a path of consecutive edges that all point in the same direction (Figure 2A). In effect, any two vertices can communicate with each other in both directions. Strong connectivity depends only on the edges and not on the labels.

A strongly connected graph can be arbitrarily large and complicated but its one-dimensional chemistry is particularly simple. The steady-state concentration of each species can be calculated in terms of the edge labels using certain sub-graphs called spanning trees (see Equation 7 below). Among other things, this shows that each microstate in a strongly connected graph has positive probability at steady state: if such a system is watched over time, each microstate will appear at steady state, even if that microstate had zero probability in the initial condition.

A general graph, which is not strongly connected, breaks up naturally into maximal strongly connected sub-graphs, or strongly connected components (SCCs) (Figure 2B). Once matter has left a SCC under one-dimensional chemistry, it can never return to it, for otherwise the SCC would not be maximal. Hence, matter eventually accumulates on those SCCs from which there is no escape, which are the terminal SCCs. If a microstate is not in a terminal SCC, its steady-state probability is zero: if the system is watched over time, such microstates never appear in the steady state, even if they had positive probability in the initial condition. For the microstates that do lie in terminal SCCs, their steady-state probability may or may not be zero depending on the initial conditions. For instance, if matter is only placed on the vertices of one terminal SCC, it will remain there forever and cannot escape into any other SCC, whose vertices will have zero probability at all times.

A system that reaches thermodynamic equilibrium always has a strongly connected graph. The property of detailed balance, which must always hold at equilibrium, requires that each edge in the graph has a corresponding reverse edge, so that strong connectivity is guaranteed. If the labels on a pair of reversible edges are *a* and *b*, then the ratio *a*/*b* is a thermodynamic quantity that depends only on the free energy difference between the two microstates (see Equation 6 below). The steady-state probabilities depend only on these thermodynamic ratios and can be calculated as products of the ratios along paths in the graph, without the need for any spanning trees (see Equation 5 below). This gives the same result as equilibrium statistical mechanics. In this way, the framework provides a generalisation of equilibrium statistical mechanics for gene-regulation systems that are far from equilibrium.

### Constructing graphs to describe gene regulation

Linear framework graphs are constructed from labelled edges, which arise from two kinds of transitions, as listed below. The main restrictive assumptions concern the interplay between mechanisms taking place in solution around chromatin and those taking place on chromatin itself. The basic approach is to assume that these can be uncoupled from each other. More relaxed assumptions can be made, using the methods of [35], but at the expense of considerably increased complexity.

#### Binding transitions

**Figure 3:**
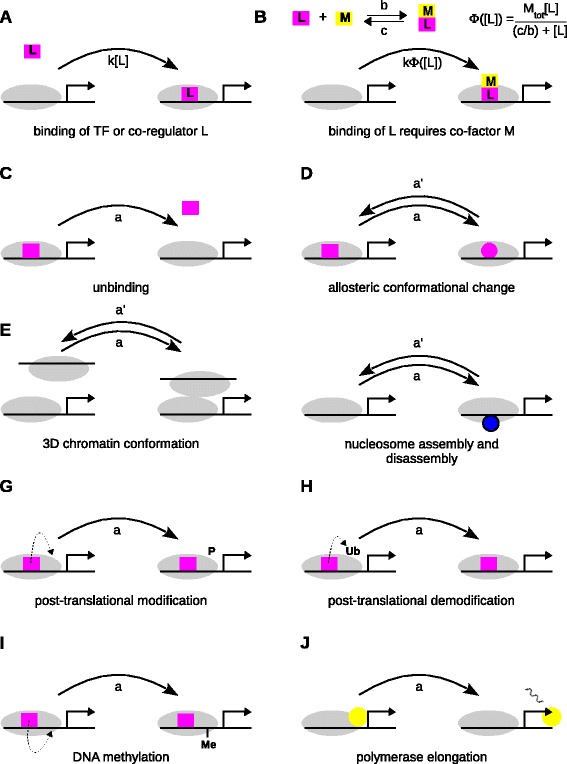
**Labelled, directed edges for graphs.** **(A, B)** Binding transitions. **(C–J)** Non-binding transitions. Each example shows a source (left) and a target (right) microstate connected by a labelled edge (curved, barbed arrow). Grey ovals signify background components that make up the microstate. A nominal transcription start site is shown. The magenta shape in **(C)**, **(D)**, **(G)**, **(H)** and **(I)** depicts a component of the source microstate that is specifically involved in the reaction represented by the edge. A small dashed arrow signifies an enzymatic action by a component in the source microstate (magenta shape), which remains bound after catalysis. The yellow disc depicts RNA polymerase with a nascent mRNA molecule in the elongating state. The edge-label formula in **(B)** comes from the rapid equilibrium assumption discussed in the text and is derived in the Methods. 3D, three dimensional; TF, transcription factor; Me, methylation; P, phosphorylation; Ub, ubiquitination.

These represent the binding of a component *L* to a microstate (Figure 3A). The label is *a*=*k*[ *L*], where *k* is an on-rate and [ *L*] is the free concentration of *L*. We follow the thermodynamic formalism and assume, first, that components are neither synthesised nor degraded over the timescale of interest so that their total amounts are conserved quantities and, second, that the depletion of *L* can be ignored, so that the binding of a single molecule of *L* does not appreciably change its free concentration, [ *L*]. In other words, [ *L*]≈*L*_tot_. Non-specific binding to DNA can significantly reduce the free concentration and if this is thought to jeopardise the no-depletion assumption, a more elaborate analysis is needed [36,44].

Components can also engage in interactions such as oligomerisation. We again follow the thermodynamic formalism and assume that such reactions are fast compared to binding reactions on DNA, so that they have reached a rapid equilibrium. The label on the edge has the form *a*=*k*[ *X*], were *k* is an appropriate on-rate and *X* is the component form that binds to DNA (Figure 3B). [ *X*] can be calculated in terms of the concentrations of the underlying components using the rapid equilibrium assumption (Methods).

#### Non-binding transitions

These are transitions in which the edge label does not contain a concentration term. They can arise from several different types of biochemical process:

- unbinding reactions, in which a component that had previously bound to form the source microstate unbinds, with the off-rate as the label (Figure 3C);
- allosteric change, in which the conformational state of DNA, or of a component or complex in the microstate, is altered (Figure 3D);
- three-dimensional chromatin conformation change, such as DNA looping, in which separate parts of a microstate, such as a distal enhancer and a proximal promoter, bind or unbind from each other (Figure 3E), with the respective rate constants as the labels;
- nucleosome assembly or disassembly, with the nucleosomes treated, for example, as individual entities (Figure 3F), so that the labels are the aggregated overall rates of the assembly or disassembly pathway;
- enzymatic activity, in which an enzyme, which is assumed to be already bound in the source microstate, undertakes a biochemical reaction that alters the microstate, such as post-translational modification or demodification of a histone, a co-regulator or a transcription factor (Figure 3G, H), or methylation or demethylation of DNA (Figure 3I, demethylation is not shown), with the enzyme catalytic rate as the label;
- RNA polymerase activity, including transcription initiation, open complex formation, promoter clearance, elongation, pausing, etc.; Figure 3J shows elongation as a single step following initiation but this can be broken down to a finer granularity as required.

Numerical values for the parameters appearing in the labels can sometimes be estimated from experimental data [10,12,45]. One of the advantages of the framework is that calculations can be undertaken with symbolic parameters, without having to know numerical values in advance.

### Calculating microstate probabilities at steady state

The mathematical details of the linear framework were developed in previous work [35-37], as reviewed in [38]. As this may not be familiar, and to keep this paper as self-contained as possible, the material is summarised here. Proofs of most of the assertions can be found in [37]. A graph of the kind constructed above, as in Figure 1, gives rise to a linear differential equation that describes how the probabilities of each microstate change in time. We first explain how this differential equation arises and then show how microstate probabilities can be calculated at steady state. The key formulas for the microstate probabilities are Equation 5 at equilibrium and Equation 7 away from equilibrium. We have italicised mathematical concepts that may be unfamiliar and have provided a glossary to explain these in the Methods.

#### Laplacian dynamics

**Figure 4:**
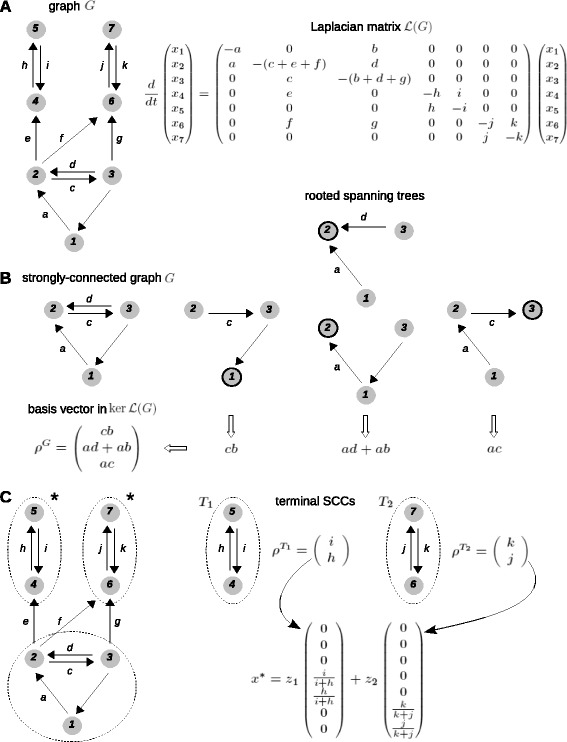
**Calculating microstate probabilities at steady state.** **(A)** On the left, a labelled, directed graph *G*; on the right, the linear differential equation obtained by taking each edge to be a chemical reaction under mass-action kinetics with the edge label as the rate constant. The resulting matrix is the Laplacian matrix, $\mathcal {L}(G)$, of *G*. **(B)** Illustration of Equation 7. On the left, a strongly connected graph; on the right, the spanning trees of the graph, each rooted at the circled vertex. Because the graph is strongly connected, each vertex has at least one spanning tree rooted there. The basis vector $\rho ^{G} \in \ker \mathcal {L}(G)$ is calculated from the spanning trees using Equation 7. Probabilities of microstates are then given by normalising the entries of *ρ* ^*G*^, as in Equation 4. **(C)** On the left, the non-strongly connected graph in **(A)** is shown along with its three strongly connected components (SCCs) demarcated by the dotted lines. The two terminal SCCs are marked with an asterisk and denoted *T*_1_ and *T*_2_. Each terminal SCC gives rise to a basis vector in $\ker \mathcal {L}(G)$ using Equation 7, as in **(B)**, and then forming a normalised vector, as shown by following the curved arrows. Note that vertices that are not in a terminal SCC (i.e., vertices 1, 2 and 3) have zero entries in each basis vector. Any steady state, *x* ^∗^, can be expressed as a linear combination of these basis vectors, as in Equation 9 SCC, strongly connected component.

Suppose we are given a graph *G*, as in Figure 4A, with vertices indexed 1,…,*n*. We typically use the index 1 for the reference microstate with no TFs bound and choose the order of the other microstates arbitrarily. The notation $i\overset {a}{\rightarrow }j$ signifies the edge with label *a* from source vertex *i* to target vertex *j*. A dynamics can be imposed on *G* in two equivalent ways. In the macroscopic interpretation, the vertices are chemical species and the edges are chemical reactions, which convert source species to target species. The edge labels are rate constants for the corresponding reactions, assuming mass-action kinetics. Since each reaction is uni-molecular, with only one substrate and one product, this one-dimensional chemistry yields a linear dynamics (Figure 4A),

(1) $$ \frac{d}{dt}x(t) = \mathcal{L}(G)\cdot x(t),   $$

where *x*(*t*) is a column vector of species concentrations and $\mathcal {L}(G)$ is an *n*×*n* matrix whose entries are labels, which is called the Laplacian matrix of *G*.

Since the dynamics inter-converts between species and neither creates matter nor destroys it, the total concentration does not change over time. The dynamics therefore satisfies the conservation law

(2) $$ x_{1}(t) + \dots + x_{n}(t) = u_{\text{tot}} \,.   $$

This corresponds to the columns of the Laplacian matrix adding up to 0 (Figure 4A), so that $1^{t} \cdot \mathcal {L}(G) = 0$, where 1 signifies the all-ones column vector and ^*t*^ denotes the transpose operation, which turns a column vector into a row vector.

In the microscopic interpretation, the vertices are microstates, the edges are transitions between microstates and the labels are *infinitesimal transition rates* for the corresponding edges. This means that, if $i\overset {a}{\rightarrow }j$ and *Δt* is a time interval sufficiently small so that *aΔt*<1, then the probability of taking the transition from state *i* to state *j* is approximately *aΔt* and the approximation gets better as *Δt* gets smaller (see Equation 15 in the glossary). This interpretation defines a continuous time, finite state *Markov process*. A Markov process gives rise to a *master equation* that describes how the microstate probabilities change over time. This master equation is identical to Equation 1, so that

$$ \frac{d}{dt}u(t) = \mathcal{L}(G) \cdot u(t), $$

where *u*_*i*_(*t*) is the probability of occurrence of microstate *i* at time *t*. The only difference with the macroscopic interpretation is that probabilities must always add up to 1, so that *u*_tot_=1 in Equation 2. Matrices of Laplacian type often arise when master equations are used but the underlying graph, from which the Laplacian can always be derived, has not been exploited as we do here.

#### Steady states

In the macroscopic interpretation, no matter what graph and what initial condition are chosen, the dynamics always reaches a steady state, *x*^∗^, in which production and consumption of each species is exactly balanced, so that, *dx*^∗^/*dt*=0. By Equation 1, *x*^∗^ is in the *kernel* of the Laplacian matrix: $x^{*} \in \ker \mathcal {L}(G)$.

A particularly important case arises when *G* is *strongly connected* (Figures 2A and 4B) because the kernel of the Laplacian is one dimensional:

(3) $$ \dim\ker\mathcal{L}(G) = 1.   $$

In other words, there is a unique steady state, up to a scalar multiple. Given a basis vector for the kernel, $\rho ^{G} \in \ker \mathcal {L}(G)$, it then follows from Equations 2 and 3 that the steady-state probabilities are obtained by normalising the entries of *ρ*^*G*^ to its total amount, ${\rho ^{G}_{1}} + \dots + {\rho ^{G}_{n}} = 1 \cdot \rho ^{G} $, so that

(4) $$ u^{*} = \left(\frac{\rho^{G}}{1 \cdot \rho^{G}}\right).   $$

Such a basis vector *ρ*^*G*^ can be constructed in one of two ways, described next.

#### At thermodynamic equilibrium

If the graph represents a system that can reach thermodynamic equilibrium, then detailed balance must be satisfied [36]. This requires two conditions to hold. First, the graph must be reversible: if the graph has an edge $i\overset {a}{\rightarrow }j$, then it must also have a reverse edge, $j\overset {b}{\rightarrow }i$, corresponding to the same underlying biochemical reaction working in reverse. Note that reversible edges imply that the graph is strongly connected. Second, in any steady state, *x*^∗^, any such pair of reversible edges must be independently at equilibrium, with the forward flux in balance with the reverse flux, irrespective of any other edges involving *i* and *j*. Setting the two fluxes to be in balance, it follows that $x^{*}_{j} = (a/b)x^{*}_{i}$.

To determine ${\rho ^{G}_{j}}$, choose any path of reversible edges from vertex 1 to vertex *j*,

$$\begin{aligned} 1 &= i_{1} \mathop{\rightleftharpoons}\limits^{a_{1}}_{b_{1}} i_{2} \mathop{\rightleftharpoons}\limits^{a_{2}}_{b_{2}} \dots \mathop{\rightleftharpoons}^{a_{p-1}}_{b_{p-1}} i_{p} \mathop{\rightleftharpoons}^{a_{p}}_{b_{p}} i_{p+1} = j, \end{aligned} $$

and let ${\rho ^{G}_{j}}$ to be the corresponding product of label ratios,

(5) $$ {\rho^{G}_{j}} = \left(\frac{a_{p}}{b_{p}}\right)\left(\frac{a_{p-1}}{b_{p-1}}\right) \dots \left(\frac{a_{2}}{b_{2}}\right)\left(\frac{a_{1}}{b_{1}}\right).   $$

It follows from detailed balance that $x^{*}_{j} = {\rho ^{G}_{j}}x^{*}_{1}$, so that *x*^∗^=*λρ*^*G*^ where $\lambda = x^{*}_{1}$. Hence, *ρ*^*G*^ provides the required basis vector of $\ker \mathcal {L}(G)$, from which probabilities can be calculated using Equation 4. For this procedure to be consistent, ${\rho ^{G}_{j}}$ must be independent of the chosen path from 1 to *j*. This is ensured by the *cycle condition*, which is a necessary consequence of detailed balance [36]. It is an important feature of being at thermodynamic equilibrium that history does not matter: any path to a microstate can be used to determine its equilibrium probability.

Equation 5 is equivalent to the thermodynamic formalism through van’t Hoff’s formula. If $i\overset {a}{\rightarrow }j$ and $j\overset {b}{\rightarrow }i$, then, at thermodynamic equilibrium,

(6) $$ \left(\frac{x^{*}_{j}}{x^{*}_{i}}\right) = \left(\frac{a}{b}\right) = \exp\left(\frac{-\Delta G}{RT}\right),   $$

where *ΔG* is the free-energy difference between microstates *j* and *i*, *R* is the molar Boltzmann constant and *T* is the absolute temperature. The product of label ratios in Equation 5 is transformed, through the exponential function in Equation 6, into a sum of free energies, which determines the free energy of microstate *j* relative to that of the reference microstate 1. The denominator in Equation 4 is then the partition function of equilibrium statistical mechanics.

**Figure 5:**
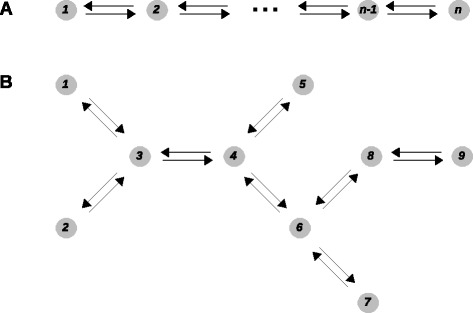
**Graph structures satisfying detailed balance.** Labels have been omitted for clarity. **(A)** A sequence of reversible edges, as considered by Ong *et al.* [46]. **(B)** A tree of reversible edges. A tree is characterised by having no cycle of reversible edges and is an example of a general graph structure that always satisfies detailed balance, irrespective of the kinds of edges in the graph and the labels on these edges (Methods).

Thermodynamic equilibrium requires detailed balance but a graph can satisfy detailed balance without being at equilibrium. For instance, certain graph structures in which each edge is reversible, such as a *sequence* structure (Figure 5A) or, more generally, a *tree* structure (Figure 5B), always satisfy detailed balance (Methods). In such a graph the edges may involve dissipative mechanisms. However, although an edge $i\overset {a}{\rightarrow }j$ is accompanied by a reverse edge $i\overset {a}{\rightarrow }j$, these edges may not arise from an underlying biochemical reaction operating reversibly but from two separate dissipative reactions, such as phosphorylation and dephosphorylation, each acting irreversibly. The ratio *a*/*b* would no longer have a thermodynamic interpretation in terms of a free energy difference, as in Equation 6.

#### Away from equilibrium

If the graph represents a system that is maintained away from thermodynamic equilibrium, then detailed balance may no longer hold. The graph may have irreversible edges and Equation 5 no longer works. If the graph is strongly connected, a basis vector of $\ker \mathcal {L}(G)$ can be calculated by the matrix-tree theorem, a proof of which is given in the Appendix to [37]. This leads to the following procedure. Let *Θ*_*j*_(*G*) be the set of *spanning trees* of *G* that are *rooted* at microstate *j*. Informally, a tree is a sub-graph with no cycles, it is spanning if it reaches every vertex and it is rooted at vertex *i* if *i* has no outgoing edges in the tree. Figure 4B gives examples of rooted spanning trees. It is not difficult to see that a graph is strongly connected if, and only if, it has a spanning tree rooted at each vertex and that a spanning tree always has one less edge than the number of vertices in *G*.

For a strongly connected graph, ${\rho ^{G}_{j}}$ may be calculated by multiplying together the labels on the edges of each spanning tree rooted at *j* and adding up these products over all such spanning trees:

(7) $$ {\rho^{G}_{j}} = \sum_{T \in \Theta_{j}(G)}\left(\prod_{k\overset{a}{\rightarrow}l \in T} a \right) \,.   $$

Because a strongly connected graph has at least one spanning tree rooted at each vertex, each entry in the basis vector is positive, so that ${\rho ^{G}_{j}} > 0$ for each *j*. Hence, by Equation 4, each microstate has positive steady-state probability. The denominator in Equation 4 provides a non-equilibrium partition function.

#### Non-strongly connected graphs

**Figure 6:**
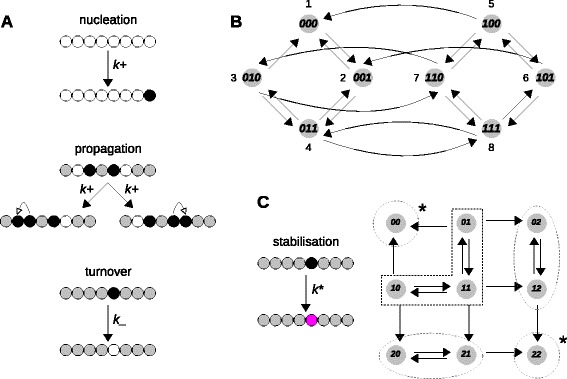
**Formation of an inherently bounded chromatin domain [** 47,48 **].** **(A)** An array of nucleosomes is shown, with nucleation taking place at the right-hand end. White nucleosomes are unmarked, black nucleosomes are marked and grey nucleosomes are either marked or unmarked. Nucleation, at rate *k*+, is confined to the nucleation site; propagation, also at rate *k*+, allows a marked nucleosome to propagate the mark to one of its two immediate (unmarked) neighbours; turnover, at rate *k*_, allows any marked nucleosome, including the nucleation site, to become unmarked. **(B)** Directed graph for the model with three nucleosomes. Each microstate shows its marking pattern as a bit string with 0 denoting unmarked and 1 denoting marked. The microstates are enumerated by considering the bit string as a number in base 2 notation and adding 1. The edges correspond to nucleation, propagation and turnover, as above. Labels have been omitted for clarity but an edge that increases, respectively decreases, the number of bits has label *k*+, respectively *k*_. **(C)** On the left, an extension of the model to include mark stabilisation, with a stably marked nucleosome shown in magenta. A stabilised mark is no longer subject to turnover. This leads to the non-strongly connected graph shown on the right for an array of two nucleosomes, in which the digit 2 in the microstate description signifies a stabilised mark. Edges that change digit 1 to digit 2 have label *k* ^∗^, while the other edges are labelled as in **(B)**. The strongly connected components (SCCs) are indicated by dotted outlines, with the two terminal SCCs identified by an asterisk.

Graphs arising in gene regulation may not always be strongly connected (see the section ‘Formation of an inherently bounded chromatin domain’ and Figure 6C). Steady-state probabilities for non-strongly connected graphs can be calculated by considering the SCCs of *G* (Figures 2B and 4C). The SCCs inherit connections from the underlying graph but these connections can never form a cycle, for otherwise the SCCs would collapse into each other. It is therefore possible to identify *terminal SCCs*, from which there are no outgoing connections. The terminal SCCs yield steady states in the following way.

Let *T*_1_,…,*T*_*t*_ denote the terminal SCCs. Each *T*_*k*_ is by definition strongly connected, so that it has a basis vector $\rho ^{T_{k}} \in \ker \mathcal {L}(T_{k})$, as given by Equation 7. We can now construct the vector *ρ*^*G*,*k*^ that agrees with $\rho ^{T_{k}}\phantom {\dot {i}\ !}$ on those microstates that lie in *T*_*k*_ and which is zero on all other microstates (Figure 4C). The vectors *ρ*^*G*,*k*^ provide a basis for the kernel of the Laplacian of *G*:

(8) $$ \ker\mathcal{L}(G) = \left\langle \rho^{G,1}, \dots, \rho^{G,t} \right\rangle \,.   $$

The dimension of the kernel is then *t*, the number of terminal SCCs. Note that, if *i* is any microstate that is not in a terminal SCC, then $\rho ^{G,k}_{i} = 0$ for each basis vector *ρ*^*G*,*k*^.

The *t* basis vectors in $\ker \mathcal {L}(G)$ are matched by *t* conservation laws. In contrast to Equation 2, which is the only conservation law when *t*=1, the additional conservation laws for *t*>1 depend on the structure of the graph. These additional laws can be algorithmically calculated from $\mathcal {L}(G)$.

Any steady state *x*^∗^ can be expressed as a linear combination of the basis vectors in Equation 8. If these vectors are normalised to their respective totals, then, in the resulting expression for *x*^∗^,

(9) $$ x^{*} = z_{1} \left(\frac{\rho^{G,1}}{1 \cdot \rho^{G,1}}\right) + \dots + z_{t} \left(\frac{\rho^{G,t}}{1 \cdot \rho^{G,t}}\right),   $$

the coefficients *z*_1_,…,*z*_*t*_ are the values taken by the *t* conservation laws.

#### Calculating gene expression

In the thermodynamic formalism, a rate of gene expression, *g*_*i*_, is assumed for each microstate *i* and the overall rate is taken to be proportional to the average over the steady-state microstate probabilities $u^{*}_{i}$. This average is given by

(10) $$ g_{1}u^{*}_{1} + \dots + g_{n}u^{*}_{n}.   $$

The same procedure is used for the examples studied here but the linear framework can accommodate the irreversible dynamics of mRNA polymerase (initiation, open complex formation, promoter escape, elongation, pausing, etc.) [17,49,50], as shown in Figure 3J. The dynamics of mRNAs and proteins can also be coupled to gene regulation within a graph-theoretic formalism [41]. However, this leads to infinite graphs because the number of mRNA or protein molecules may be unlimited.

Having summarised the linear framework and shown how it generalises the thermodynamic formalism to non-equilibrium contexts, we now discuss three applications that demonstrate the framework’s scope.

### Regulation of steroid-hormone responsive genes

Ong *et al.* have put forward a theoretical framework for gene induction [46], motivated by studies of steroid-hormone receptors [51]. They use *ad hoc* methods, which are independent of previous work on gene regulation. We show here how their analysis can be generalised and simplified within the linear framework.

Recent work on steroid-hormone sensitive genes has revealed new co-regulators, such as the Ubiquitin conjugating enzyme, Ubc9, indicating the existence of multiple steps in addition to hormone-receptor binding to DNA [46]. Despite this additional complexity, gene-regulation functions [16], which describe how rates of gene expression depend on hormone concentration, are well fitted to Michaelis–Menten style functions, or first-order Hill dose–response curves (FHDCs) in the language of Ong *et al.*, who use their theoretical framework to derive conditions under which such FHDCs arise.

They consider a sequence of reversible reactions (Figure 5A), representing the behaviour of the promoter of a hormone-sensitive gene. Such a sequence graph always satisfies detailed balance (Methods). We consider the more general case of an arbitrary graph *G* of reversible edges that satisfies detailed balance. This might be, for instance, a tree graph (Figure 5B), which also always satisfies detailed balance (Methods). If a general graph satisfies detailed balance it may not necessarily reach thermodynamic equilibrium and the edges of *G* may involve dissipative mechanisms.

We assume that components *R*,*U*,*Y*_1_,…,*Y*_*m*_ are present and they can bind and unbind to form the microstates of *G*. *Y*_1_,…,*Y*_*m*_ are background components that can engage in protein–protein interactions among themselves, so that their concentrations can appear in labels of the form $k\Phi ([\!Y_{i_{1}}], \dots, [\!Y_{i_{k}}]\!)$, where *Φ* is some function, as in Figure 3B. The no-depletion assumption allows free concentrations to be replaced by total concentrations, [ *Y*_*i*_]≈ *Y*_*i*,tot_, so that the labels in which *Y*_1_,…,*Y*_*m*_ occur are functions of rate constants and total amounts, or “constants”. *R* and *U* are titratable components, which, crucially, are assumed to bind at most once in each microstate. *U* corresponds to a co-regulator like Ubc9, which does not engage in protein–protein interactions, so that the corresponding label has the form *k*^′^[ *U*] (Figure 3A). *R* corresponds to the steroid-hormone receptor, to which the steroid hormone *S* binds to form a complex *RS*, which then binds DNA (Figure 3B with *S*=*L* and *R*=*M*). The label on the corresponding edge has the form *k*^″^[ *RS*] where

$$ [\!RS] = \frac{R_{\text{tot}}[\!S]}{K_{R} + [\!S]}, $$

which is a FHDC as a function of [ *S*].

The main result is that, provided gene expression only occurs from microstates in which both *R* and *U* are bound, the average rate of gene expression, *g*([ *S*]), as given by Equation 10, is also a FHDC (Additional file 1A),

(11) $$ g([\!S]\!) = \frac{M_{G}[\!S]}{K_{G} + [\!S]} \,.   $$

The constants *M*_*G*_ and *K*_*G*_ have clear interpretations in terms of *G*. *M*_*G*_ is (evidently) the average rate of gene expression at saturation (i.e., when [ *RS*]=*R*_tot_). Less obviously, *K*_*G*_ is *K*_*R*_ multiplied by the saturation probability of those microstates in which *R* is not bound. Additional file 1A gives the details of the proof and shows how the formulas in Ong *et al.* emerge from Equation 11. It also discusses how Ong *et al.* show, for the special case of a sequence, that *g*([ *S*]) remains a FHDC even if the no-depletion assumption is dropped at a concentration limiting step. Ong *et al.* also address other issues, such as inhibitory reactions, which are not discussed here.

The framework introduced here generalises and clarifies the work of Ong *et al.*, showing how formulas like Equation 11 can be rigorously proved irrespective of the complexity of the underlying graph. The interpretation of the parameters in Equation 11 is new but emerges easily from our analysis (Additional file 1A). However, because detailed balance is assumed, the consequences of being away from equilibrium remain hidden, as we will see subsequently.

### Formation of an inherently bounded chromatin domain

Our next application is to a model of chromatin organisation, with no explicit gene regulation. Hathaway *et al.* recently showed how a bounded chromatin domain could be nucleated *in vivo* and stably inherited as a form of epigenetic memory [47]. To explain the dynamics of such domains, they developed a mathematical model based on a linear array of 257 nucleosomes [47,48]. This model is readily translated into our framework. We considered nucleosome arrays with varying numbers of sites *n*. We placed the nucleation site at the right-hand end of our array (Figure 6A). This is essentially similar to the left-hand half of the array of 2*n*−1 nucleosomes (for *n*=129) considered by Hathaway *et al*. The microstates correspond to array marking patterns, of which there are 2^*n*^, while the edges correspond to mark nucleation, propagation and turnover (Figure 6A,B). Propagation and turnover were assumed uniform at all nucleosomes, at rates *k*+ and *k*_, respectively. However, nucleation was limited to the nucleation site at rate *k*+, so that some edges are not reversible. This irreversibility reflects the dissipative mechanism of histone marking and the non-equilibrium nature of the model. The graph does not satisfy detailed balance but is strongly connected.

Hathaway *et al.* used a Monte Carlo simulation to generate stochastically a succession of microstates, from which steady-state probabilities were estimated as the frequencies with which microstates appear. They found that, if *k*+/*k*_≤1.5, marking persisted in a stochastically fluctuating but inherently bounded domain near the nucleation site, reflecting what was found experimentally.

Monte Carlo simulation is an efficient method for studying very large graphs: an array of 257 nucleosome has a graph with approximately 10^77^ microstates. However, the linear framework provides mathematical access to the steady-state probabilities for any array size and this yields insights that are not easily found by simulation. For instance, the ratio *k*+/*k*_ appears as a convenience in the simulations [48]. However, for a nucleosome array of *n* sites, the spanning trees in the corresponding graph (Figure 6A) have 2^*n*^−1 edges, each of which is labelled *k*+ or *k*_. Dividing Equation 7 by $(k\_)^{2^{n}-1}$, it is evident that the steady-state probabilities in Equation 4 depend only on the ratio *k*+/*k*_ and not on the individual rates. The importance of the ratio becomes readily apparent within our framework.

More significantly, Hathaway *et al.* proposed a modification to their model to explain the inherited stability of the domain after the nucleating stimulus was removed. They imposed a stabilisation of the nucleosome mark through a transition to a hypothetical new marked state, whose turnover was inhibited (Figure 6C, left). Each nucleosome can now be in one of three states and the graph has 3^*n*^ microstates (Figure 6C, right, for *n*=2). Because turnover is prevented by the stabilised mark, the graph is no longer strongly connected. If nucleation is stopped, as was done in the simulation, then the resulting graph has two terminal SCCs, each consisting of a single extreme microstate, one in which the entire nucleosome array is unmarked and the other in which the entire array is stably marked. According to Equation 9, all other microstates have zero steady-state probability.

Which of the two extreme microstates is reached in a simulated trajectory depends on the microstate in which nucleation is stopped. If some nucleosome has become stably marked in that microstate, then it cannot become unmarked, so the trajectory can only reach the completely stably marked microstate. This is likely to happen once the inherently bounded domain is established, unless the stabilisation rate, *k*^∗^, is so low that no stable mark has appeared. In their simulation, Hathaway *et al.* chose *k*^∗^ to be low compared to propagation and turnover but not so low that stable marks had not appeared by the time nucleation was stopped. They concluded that the inherently bounded domain was stably maintained in the absence of the initial nucleating stimulus. Our analysis shows that this conclusion is incorrect. Once nucleation is stopped, the bounded domain becomes a transient phenomenon, which eventually expands to fill the whole array. It is conceivable that a bound on the domain size is maintained for sufficiently long to still be biologically relevant. But this places the stabilising rate *k*^∗^ in a double bind: it must be sufficiently high so as to stabilise the domain, yet sufficiently low so as not to destroy its boundedness too quickly. Such fine-tuning of rate constants is inherently fragile and we think it is more likely that other mechanisms are at work to ensure stable inheritance of the inherently bounded domain.

Our framework allows these conclusions to be reached by elementary mathematical deductions, without the need for the numerical simulations undertaken by Hathaway *et al*.

### Regulation of yeast *PHO5*

**Figure 7:**
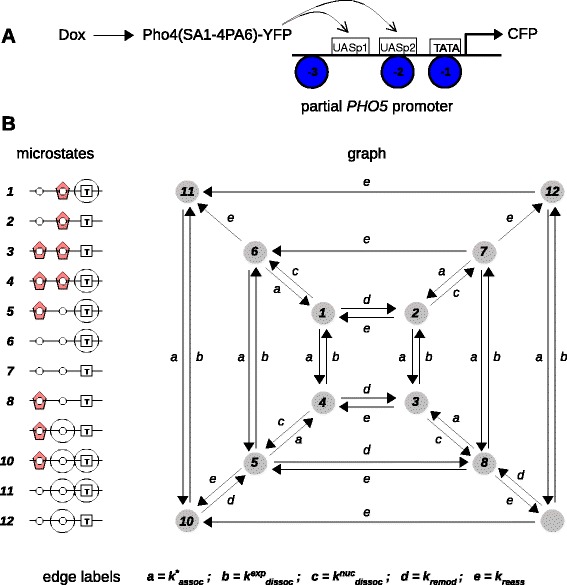
**Regulation of yeast** ***PHO5*** **, adapted from Figures one and four b of [** 52 **].** **(A)** Schematic of the experimental set-up. A doxycycline-inducible (Dox), YFP-tagged Pho4, modified to be constitutively active (SA1-4) and constitutively nuclear (PA6), stimulates expression of CFP from a partial *PHO5* promoter, with three nucleosomes (-3, -2 and -1) and two Pho4 binding sites, a low-affinity exposed site between nucleosomes -2 and -3 (UASp1) and a high-affinity site occluded by nucleosome -2 (UASp2). The TATA box is occluded by nucleosome -1. **(B)** The labelled, directed graph of this system, showing the microstates (left) and the labels (bottom), in the notation used by Kim and O’Shea. Label *a* $\left (k^{*}_{\text {assoc}}\right)$ corresponds to Pho4 binding through a Hill function, which arises through the rapid equilibrium mechanism of Figure 3B. Labels *b* $\left (k^{\text {exp}}_{\text {dissoc}}\right)$ and *c* $\left (k^{\text {nuc}}_{\text {dissoc}}\right)$ correspond to Pho4 unbinding (Figure 3C) from, respectively, UASp1 and UASp2. Labels *d* (*k*_remod_) and *e* (*k*_reass_) correspond to disassembly and assembly, respectively, of nucleosomes (Figure 3F), which introduce the non-equilibrium and irreversible features of the graph. Nucleosome -3 has been ignored in the graph. For other features, see the cited paper CFP, cyan fluorescent protein; YFP, yellow fluorescent protein.

We now turn back to gene regulation and to one of the very few models in which a non-equilibrium mechanism has been rigorously analysed without assuming detailed balance. Pho5 is an acid phosphatase in *Saccharomyces cerevisiae* that is expressed under phosphate-starvation conditions. Kim and O’Shea undertook a quantitative analysis of *PHO5* regulation by the transcription factor Pho4, using a construct detached from the phosphate-response pathway [52] (Figure 7A).

To calculate the *PHO5* gene-regulation function, Kim and O’Shea constructed a stochastic master equation based on a graph of transitions between DNA states. They pointed out that the nucleosomal transitions were dissipative and in some cases irreversible under their assumptions, so that detailed balance could not be assumed. Accordingly, they determined steady-state probabilities using the Symbolic Math Toolbox in MATLAB.

Kim and O’Shea’s graph of transitions is readily translated into our linear framework (Figure 7B). They assumed that the binding of Pho4 saturates according to a Hill function, which can be accommodated in a similar way to Figure 3B. The non-binding reactions correspond to unbinding of Pho4 (Figure 3C), or to nucleosomal assembly or disassembly (Figure 3F). The graph is strongly connected, a point not mentioned by Kim and O’Shea, but as noted above for Equation 7, this ensures that the steadystate probability of each microstate is positive. They assumed that *PHO5* is transcribed when there is no nucleosome occluding the TATA box, so that, in the average in Equation 10, *g*_*i*_=1 for the microstates 2, 3, 7, 8, 9 and 12 on the right in Figure 7B and *g*_*i*_=0 for those on the left. We used our own software written in the programming language Python to enumerate the spanning trees by a fast algorithm and then used the polynomial algebra capabilities of Mathematica to calculate the microstate probabilities and the gene-regulation function (Methods). This gave an identical result to Kim and O’Shea’s MATLAB calculation (H Kim, personal communication, January 2013). This strongly suggests that what can be done for the yeast *PHO5* gene can be systematically undertaken for other genes with non-equilibrium features, with the solution now being understood explicitly through Equation 7, without recourse to MATLAB.

Having calculated the gene-regulation function using our framework, we sought to compare it to the experimental data acquired by Kim and O’Shea [52]. They used their synthetic construct (Figure 7A, with details in the caption) to measure the *PHO5* gene-regulation function. In response to doxycycline, individual cells expressed Pho4-YFP, which was treated as the input to the gene-regulation function, and this induced the expression of CFP from the Pho4-responsive promoter in the construct. CFP was treated as the output as a proxy for Pho5. By using different doses of doxycycline to cover a range of Pho4-YFP expression levels, the gene-regulation function was assembled from single-cell measurements. Kim and O’Shea also measured the gene-regulation function of five other variant promoters, in which the low-affinity and high-affinity sites for Pho4 binding were either exchanged or removed.

**Figure 8:**
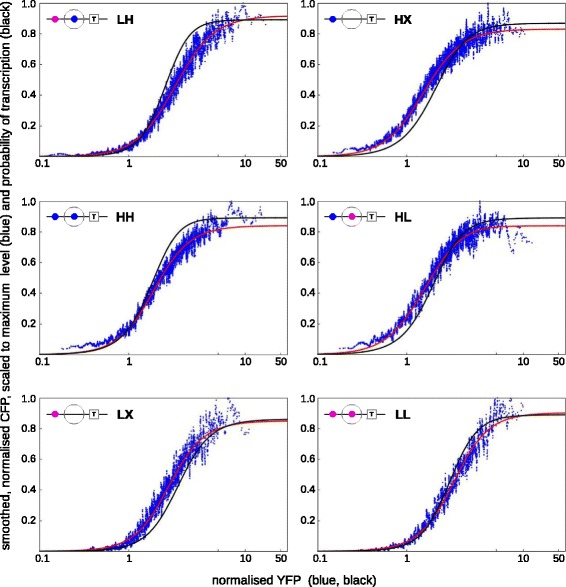
**Experimental data and calculated gene-regulation functions of** ***PHO5*** **variants.** Each panel corresponds to one of the six variants, as labelled in the top left with high affinity (H, blue), low affinity (L, magenta) or absent (X), using the microstate schematic from Figure 7B. Each panel shows the smoothed and normalised experimental data for that variant scaled to its maximum expression level (blue points) and plotted as normalised CFP for output against normalised YFP for input, overlaid with calculated gene-regulation functions for that variant (red and black curves), plotted as probability of transcription against normalised YFP, which is assumed to be proportional to Pho4 concentration. The red curves show individual fits to each variant, while the black curves show a collective fit to all variants simultaneously. Further details are provided in the text and the Methods. H, high affinity; L, low affinity; X, absent.

Kim and O’Shea estimated the threshold and the maximum expression level of each variant by fitting their experimental data to a Hill function, whose Hill coefficient was found to be nearly 2 for all variants. They then fitted the estimated threshold and maximum values to the calculated gene-regulation function for each variant and found good agreement ([52], Figure 5). We were curious as to how well the gene-regulation function itself would fit the data. This is a more challenging question because the data are noisy and the gene-regulation function is very complicated (see below). To address this, we first smoothed the data. We then used numerical optimisation to find excellent quantitative fits to each variant individually (Figure 8, red curves) but could only undertake a manual fit to all variants collectively, which yielded the parameter values in Equation 16 (Methods). The collective fit was considerably poorer (Figure 8, black curves). While this broadly confirms Kim and O’Shea’s more coarse-grained analysis, it also suggests that the individual variants may exhibit more nuanced behaviours, which are better described by distinct parameter values.

### History-dependent complexity away from equilibrium

Our analysis revealed further unexpected features of the *PHO5* gene-regulation function. By Equation 7, each ${\rho ^{G}_{i}}$ is a sum of distinct product terms (monomials) in the five edge labels (Figure 7B), of the form

$$ \alpha \left(k^{*}_{\text{assoc}}\right)^{i_{1}}\left(k^{\text{exp}}_{\text{dissoc}}\right)^{i_{2}}\left(k^{\text{nuc}}_{\text{dissoc}}\right)^{i_{3}}\left(k_{\text{remod}}\right)^{i_{4}}\left(k_{\text{reass}}\right)^{i_{5}}. $$

Here, *α* is a positive integer, which records the number of spanning trees having that product of labels, and *i*_1_,…,*i*_5_ are non-negative integers. Because the graph has 12 microstates, each spanning tree has 11 edges, so that the total degree of each monomial is 11: *i*_1_+*i*_2_+*i*_3_+*i*_4_+*i*_5_=11. By examination of the calculated formulas, the maximal degree of $k^{*}_{\text {assoc}}$, in which the concentration of Pho4 appears, is 8. Considering only those monomials with this highest-order term, $\left (k^{*}_{\text {assoc}}\right)^{8}$, the gene-regulation function looks like

(12) $$ \frac{4(k_{\text{remod}})^{2}(k_{\text{remod}}+k_{\text{reass}})(k^{*}_{\text{assoc}})^{8} + \dots}{4(k_{\text{remod}})(k_{\text{remod}}+k_{\text{reass}})^{2}(k^{*}_{\text{assoc}})^{8} + \dots}.   $$

The simplicity of these highest-order terms is deceptive, however. The numerator of Equation 12 has 261 distinct monomials while the denominator has 500 distinct monomials. Indeed, the graph in Figure 7B has 53,376 spanning trees in total. We see that the calculated *PHO5* gene-regulation function is very complicated – the full details shown in Additional file 1C cover six pages – despite the model having only two binding sites and two nucleosomes. Because Kim and O’Shea did not provide the gene-regulation function in their original paper, these features are revealed here for the first time.

The linear framework allows us to understand this surprising explosion in complexity. At equilibrium, Equation 5 shows that any single path to a microstate can be used to calculate its steady-state probability. As a physicist would say, free energy at equilibrium is a function of the microstate, not of the route through which that microstate is reached. In marked contrast, away from equilibrium, Equation 7 shows that every spanning tree rooted at that microstate is required. In this case, all routes to the microstate become relevant and microstate probabilities depend in a more intricate way on the structure of the graph. Equation 7 takes care of the bookkeeping. The number of spanning trees increases very rapidly with the size of a graph: the complete undirected graph on *n* vertices (i.e., the graph in which there is an undirected edge between each pair of distinct vertices) has *n*^*n*−2^ spanning trees in total. This worse than exponential increase manifests itself in the complexity of the *PHO5* gene-regulation function.

It is important to appreciate, however, that it is not the complexity or the size of a graph that is the dominant factor in explaining the complexity found here. If we imposed additional edges on the graph in Figure 7B so as to make all the edges reversible, this would only make the graph more complex. If we then imposed detailed balance, which restricts the values of the parameters, the equilibrium probabilities would be given by Equation 5 rather than Equation 7 and the gene-regulation function could be written down in a few lines. The complexity uncovered here depends crucially on being far from thermodynamic equilibrium.

Additional study of *PHO5* has shown that nucleosomes decouple the threshold for *PHO5* expression from its dynamic range [53]. However, this kind of behaviour can be recapitulated within the thermodynamic formalism [54]. This suggests that the full implications of non-equilibrium behaviour, as revealed by the complexity of the *PHO5* gene-regulation function, have not yet been uncovered experimentally. To suggest experimental options, we need ways to decompose the complexity found in Additional file 1C and to attribute aspects of it to specific biochemical mechanisms. Approximation methods may help in particular cases [55] but new ideas are needed for addressing the complexity barrier systematically, to which we now turn.

### Graph independence leads to reduced complexity

Gene regulation often takes a modular form, with repeated binding sites, reiterated motifs and multiple enhancers [56,57]. The microstate probabilities and the resulting gene-regulation function could become extremely complicated, especially if the modules are operating far from equilibrium. There is, however, one context in which simplification may be expected. This occurs when modules operate independently of each other, so that whatever takes place within one module does not affect what takes place in any other module. For instance, developmental genes are often regulated by multiple enhancers, which sometimes appear to act independently of each other [58].

Within the thermodynamic formalism, independence of binding sites leads to multiplication of the corresponding partition functions (described after Equation 6). For instance, a transcription factor, *T*, binding to a single site on DNA has the partition function 1+*K*[ *T*], where *K* is the association constant for binding. Suppose that there are *m* repeated binding sites to which *T* binds and suppose that each site has the same association constant. If these bindings are independent of each other, then the partition function for the *m*-site system is obtained by simply multiplying the one-site partition function *m* times, to yield

(13) $$ \vspace*{-12pt}(1 + K[\!T]\!)^{m} \,.   $$

On the other hand, if the sites are not independent, the partition function takes the more complicated form

$$\begin{aligned} 1 &+ a_{1}K[\!T] + \,a_{2}(K[\!T]\!)^{2} + \dots + a_{m-1}(K[\!T]\!)^{m-1}\\ &+ a_{m}(K[\!T]\!)^{m}, \end{aligned} $$

where *a*_1_,…,*a*_*m*_ can be arbitrary numbers. Evidently, the partition function in Equation 13 is considerably less complex and easier to understand. In the light of this result for equilibrium systems, we wanted to find a generalisation in which the modules are no longer individual binding sites but are represented by potentially complex graphs, which may not be at thermodynamic equilibrium. Such modules might correspond, for instance, to independent enhancers.

**Figure 9:**
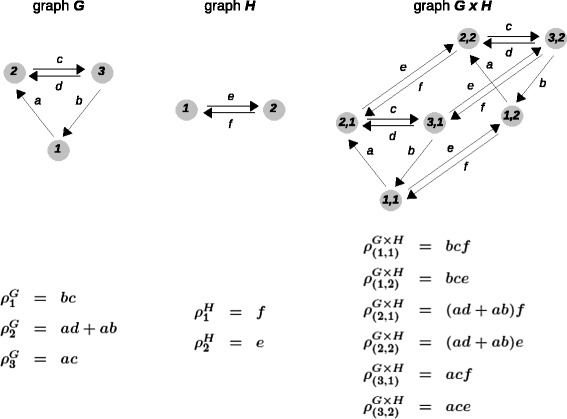
**The product graph construction.** The corresponding basis vector in the respective Laplacian kernel is shown below each graph. For legibility, the vertices of the product graph are denoted *i*,*j*, rather than (*i*,*j*). All three graphs are strongly connected. The basis vector for the Laplacian kernel of graph *G* was calculated in Figure 4B, while that for graph *H* follows directly from Equation 7. The basis vector for the Laplacian kernel of *G*×*H* is given by the Kronecker product formula in Equation 14, as described in the text.

We used the product graph construction to capture the concept of independence. Let *G* and *H* be any two graphs which represent two modules within a gene regulation system. We make no assumptions about the graphs, which do not have to be at equilibrium and do not have to be strongly connected. The product graph *G*×*H* is constructed as follows (Figure 9). It has vertices (*i*,*j*), where *i* is a vertex in *G* and *j* is a vertex in *H*. The vertices are enumerated lexicographically, so that (*i*,*j*)<(*i*^′^,*j*^′^) if either *i*<*i*^′^ or *i*=*i*^′^ and *j*<*j*^′^. For each labelled edge $i_{1}\overset {a}{\rightarrow }i_{2}$ in *G* and for every vertex *j* in *H*, the labelled edge $(i_{1},j)\overset {a}{\rightarrow }(i_{2},j)$ is created in *G*×*H*. The retention of the same label *a* on these edges ensures that the transition from (*i*_1_,*j*) to (*i*_2_,*j*) occurs independently of *j* and always at the same rate, which captures the independence assumption. Similarly, for each labelled edge $j_{1}\overset {a}{\rightarrow }j_{2}$ in *H* and for every vertex *i* in *G*, the labelled edge $(i,j_{1})\overset {b}{\rightarrow }(i,j_{2})$ is created in *G*×*H*. These are the only edges in *G*×*H*.

If the modules represented by *G* and *H* are operating independently of each other, then the graph of the combined system is given by *G*×*H*. What can be said about the *ρ*^*G*×*H*^ in terms of *ρ*^*G*^ and *ρ*^*H*^? When *G* and *H* are both strongly connected, then *G*×*H* is also strongly connected and a basis vector in the kernel of the Laplacian is given by

(14) $$ \rho^{G \times H} = \rho^{G} \otimes \rho^{H} \,.   $$

This uses the Kronecker product of two vectors, *x*⊗*y*, defined by (*x*⊗*y*)_(*i*,*j*)_=*x*_*i*_*y*_*j*_ (Figure 9). If either *G* or *H* are not strongly connected then *G*×*H* will not be strongly connected. A basis for the Laplacian kernel of *G*×*H* is then given by the Kronecker products *ρ*^*G*,*i*^⊗*ρ*^*H*,*j*^ between each pair of basis vectors from each respective kernel. The precise product theorem is stated and proved in Additional file 1B.

In the example in Figure 9, the product theorem yields polynomials for the components of *ρ*^*G*×*H*^ that have degree 3 in the labels. Since *G*×*H* is strongly connected, *ρ*^*G*×*H*^ can also be calculated using the matrix-tree formula in Equation 7. The resulting polynomials must have degree 5 because *G*×*H* has six vertices. However, each of the polynomials from Equation 7 has the same scalar factor of degree 2, given by

$$ b (c + e + f) + (e + f) (c + d + e + f) + a (b + c + d + e + f), $$

which can be divided out to give the much simpler expressions in Figure 9. The basis vectors from the product theorem are substantially less complicated, both in degree and in the numbers of monomials, than those from Equation 7.

This product theorem is important because it shows that a system that is far from equilibrium may still have simple expressions for its microstate probabilities. What is required is that the system has independent modules within it. This suggests a starting point for addressing the complexity challenge identified above, as reviewed further in the Discussion below.

## Discussion

The equilibrium thermodynamic formalism has been widely adopted and has been very effective, as reviewed in [15-19]. The value of the new framework introduced here rests on extending this to accommodate non-equilibrium, dissipative mechanisms. Although life itself is fundamentally dissipative – we are only at equilibrium when we are dead – and the importance of dissipation has been broadly understood at the molecular level [25], its significance for gene regulation has remained elusive.

Recent work has started to reveal the limitations of equilibrium assumptions. Gelles and colleagues, using single-molecule methods on *E. coli* promoters, assert that ‘it may be necessary to consider that transcription output is a non-equilibrium phenomenon controlled by the kinetic properties of the system, not simply its thermodynamics’ [22]. Lieb and colleagues, using a genome-wide competition ChIP assay in yeast, show that thermodynamic quantities are substantially less well correlated with gene expression than kinetic quantities [23]. Reviewing these and other developments, Larson and colleagues state that: ‘Currently, most quantitative theoretical models describe transcriptional regulation as an equilibrium thermodynamic phenomenon.... Here we explain how this description is fundamentally inconsistent with the canonical view of gene regulation’ [24].

Despite these assertions, no specific information-processing task has been identified that cannot be achieved at equilibrium and for which non-equilibrium mechanisms are essential. We can suggest three possibilities where that might be the case.

First, the experimental construction of an inherently bounded chromatin domain by Hathaway *et al.* relies on irreversible, dissipative mechanisms. If their model is forced to be at equilibrium by imposing reversibility of the edges, it can be readily seen that the inherently bounded domain vanishes (Methods). This suggests that dissipation is essential for maintaining a bounded chromatin domain.

Second, recent work indicates that nucleosome positioning may depend crucially on non-equilibrium mechanisms. It has been suggested that both the SWI/SNF and ISWI/ACF chromatin remodelling complexes use an ATP-dependent kinetic proofreading scheme to find the correct nucleosomal substrates on which to act [59,60], in a manner essentially identical to Hopfield’s original scheme [61]. In contrast, as mentioned in the Background, nucleosomes have been treated as competing with transcription factors for binding to DNA within the thermodynamic formalism, ignoring the dissipative aspects [18,62]. In support of this, Segal and Widom pointed out that *in vitro* reconstitution experiments using purified histones and genomic DNA, which would be expected to reach equilibrium, reproduce many aspects of *in vivo* nucleosome organisation. However, it has been a matter of contention as to how closely *in vivo* nucleosome organisation is matched *in vitro*. In attempting to resolve these issues, Struhl and Segal [21] point to more recent work [20] in which reconstitution with whole-cell extract and ATP, presumably involving ATP-dependent nucleosome remodellers, significantly improves *in vitro* recapitulation. Genetic deletion of nucleosome remodellers also has distinctive effects on nucleosome organisation. Pugh and colleagues suggest, in contrast to Segal and Widom, that ‘the active nucleosome organization *in vivo* may be at steady state, under the continuous expense of energy, rather than at equilibrium’ [20].

Third, we suggest that the combination of developmental precision and evolutionary plasticity may require non-equilibrium mechanisms. Experimental studies of the early *Drosophila* embryo suggest that the precision with which the *hunchback* gene is turned on and off in individual cells, in response to the maternal morphogen Bicoid, is close to the limits set by physics [63]. Nevertheless, the *hunchback* promoter varies considerably in the numbers and the positions of Bicoid binding sites between different species of Diptera [64], suggesting high evolutionary plasticity. While it may be possible to construct equilibrium mechanisms that achieve high precision, it seems difficult to achieve plasticity also. We speculate that non-equilibrium mechanisms may be essential to achieve both.

The framework that we have introduced here provides the foundation from which to explore such possibilities systematically. It has revealed the profound difference between equilibrium and non-equilibrium mechanisms, prefigured in Hopfield’s earlier work [25], but the remarkable complexity that we have uncovered away from equilibrium presents a formidable challenge. This complexity is fundamental because it arises from the underlying physics: history cannot be ignored away from thermodynamic equilibrium. We see two strategies for addressing this.

First, one strand of research within non-equilibrium statistical mechanics has sought to clarify the relationship between thermodynamic forces and microscopic fluxes within a graph-theoretic formalism [65] (further historical connections are reviewed in [37]). More recent developments in non-equilibrium statistical mechanics [66,67] may help to decompose the history-dependent complexity into physically meaningful components, which may then be experimentally accessible.

Second, from a mathematical perspective, our work shows that the complexity is modulated by the structure of the graph. Independence decreases the complexity, as in Figure 9, as does equilibrium, as in Equation 5. It may be reasonable to assume that some parts of a graph are at equilibrium, with dissipation serving not to maintain these microstates but, rather, to provide access to them over energy barriers, as previously suggested by Segal and Widom for nucleosome positioning [18], while other parts of the graph are maintained far from equilibrium and yet other parts may operate independently. If we could understand how to partition graphs in this way and how such partitioning simplified the steady-state probabilities, then we might have a means to address the complexity problem. We plan to explore these strategies in subsequent work. We anticipate that an inter-disciplinary approach, combining biological experiments with physics and mathematics, will be essential to unravel how graph structure gives rise to function in the context of gene regulation.

A flood of new information about nucleosome positions, histone marks and DNA methylation is emerging from whole-genome projects such as ENCODE [28], the NIH Roadmap Epigenomics Project [29] and the European BLUEPRINT project [30]. The thermodynamic formalism has been successfully applied to whole-genome analysis at single-base pair resolution. The corresponding graphs are even larger than those arising in Hathaway *et al.*’s study of bounded chromatin domains, with 10^77^ vertices, yet powerful dynamic programming methods allow equilibrium probabilities to be estimated from data [10,12]. Incorporating non-equilibrium mechanisms on a whole-genome basis may be currently infeasible but similar approximation methods could plausibly be applied to individual genes, for which information may be available on how different molecular mechanisms interact, allowing the structure of the graph to be exploited, as suggested above, to reduce the complexity. We envisage, in this way, that the function of individual genes will come to be represented by mathematical graphs, just as the structure of individual genes has been represented by mathematical sequences. In contrast to sequences, graphs encode dynamics and functionality and their structures will change with our assumptions and data. Our existing sequence-based computational infrastructure may have to evolve to an infrastructure in which such dynamic graphs can be built, interrogated and analysed.

## Methods

The experimental data discussed in this paper were obtained solely from the literature.

### Calculating labelling functions

Figure 3B shows a sequence-specific transcription factor *L* that binds DNA only when also bound to a co-factor *M*. The component form that binds to DNA (which was called *X* in the main text) is *LM*. The rate constant for the transition is proportional to the free concentration of *X*=*LM*. This free concentration can be calculated by assuming that the binding of *L* and *M*,

$$ L + M \overset{b}{\underset{c}\rightleftharpoons} L\!M $$

has reached a rapid equilibrium, independently of the binding of *LM* to DNA. In this case, *b*[ *L*][ *M*]=*c*[ *LM*], so that

$$\begin{aligned} M_{\text{tot}} &= \,[\!M] + \,[\!LM] = \,[\!M] + \,(b/c)[\!L][\!M]\\ &= (1 + (b/c)[\!L])[\!M] \,. \end{aligned} $$

It follows that

$$[\!LM] = (b/c)[\!L][\!M] = \frac{(b/c)M_{\text{tot}}[\!L]}{1 + (b/c)[\!L]} = \frac{M_{\text{tot}}[\!L]}{(c/b) + \,[\!L]}, $$

which gives the formula for *Φ*([ *L*]) shown in Figure 3B. Rapid equilibrium amounts to a timescale separation, which uncouples the dynamics of the interactions in solution from those on DNA. The rapid equilibrium equations for more complicated interactions can often be formulated in terms of the linear framework, which can then be used to calculate [ *X*].

### Glossary of mathematical concepts

*Markov process.* A time-varying probability distribution over a set of states in which the probability of reaching a given state in the next time step depends only on the current state. If time varies continuously then the next time step is interpreted infinitesimally, by taking a small unit of time, *Δt*, and letting this tend to zero. The Markov property says that history does not matter in making the choice of which state comes next in time. However, history may be essential for determining the steady-state probabilities, as happens when the system is far from thermodynamic equilibrium.

*Infinitesimal transition rate.* Suppose that $i\overset {a}{\rightarrow }j$ is a labelled, directed edge in the graph. Treating the labels as infinitesimal transition rates defines a continuous-time, finite state Markov process, *X*(*t*), as follows: in any sufficiently small unit of time, *Δt*, the conditional probability of microstate *j* occurring, given that microstate *i* has occurred, is *aΔt*, to first order in *Δt*. More formally,

(15) $$ {\lim}_{\Delta t \rightarrow 0}\frac{\text{Pr}(X(t+\Delta t) = j \,|\, X(t) = i)}{\Delta t} = a \,.   $$

With this notation, the probability of occurrence of microstate *i* at time *t*, which was denoted *u*_*i*_(*t*) in the main text, is given by *u*_*i*_(*t*)=Pr(*X*(*t*)=*i*).

*Master equation.* The probability of being in microstate *i* at time *t*+*Δt*, *u*_*i*_(*t*+*Δt*), can be calculated in terms of *u*_*j*_(*t*) and the infinitesimal transition rate from *j* to *i*, taking into account all microstates *j* that have an edge to *i*. The resulting differential equation, obtained by letting *Δt*→0, which describes the forward evolution of probabilities over time, is the master equation, or Kolmogorov forward equation, of the Markov process [68]. The equivalence between the master equation of *X*(*t*) and Laplacian dynamics is proved in ([37], Corollary 2).

Kernel. If *M* is an *n*×*n* matrix acting on column vectors of size *n*, then the kernel of *M*, ker*M*, is the subspace of column vectors that become zero when multiplied by *M*: ker*M*={*v* | *M*·*v*=0}.

Strongly connected. In a graph *G*, vertex *i* is said to ultimately reach vertex *j*, denoted *i*⇝*j*, if either *i*=*j* or there is a path of directed edges from *i* to *j*:

$$ i = i_{1} \rightarrow i_{2} \rightarrow \dots \rightarrow i_{m-1} \rightarrow i_{m} = j. $$

Vertex *i* is said to be strongly connected to *j* if *i*⇝*j* and *j*⇝*i*. Strong connectivity is an equivalence relation on the vertices and the equivalence classes are called the SCCs of *G*. A graph is strongly connected if it has only one SCC. The graph in Figure 4B is strongly connected.

Cycle condition. If a graph describes a system that can reach thermodynamic equilibrium then it must satisfy detailed balance, as described in the main text. If detailed balance holds, then, in any cycle of reversible edges, the product of the labels going clockwise around the cycle must equal the product of the labels going counterclockwise around the cycle. Conversely, if a graph has reversible edges and the cycle conditions holds, then detailed balance is satisfied for any steady state of the graph. This is proved in ([36], Supporting Information).

Sequence/tree of reversible edges. A graph consisting of reversible edges, which are arranged in a sequence (Figure 5A) or, more generally, in a tree structure (Figure 5B), automatically satisfies detailed balance, irrespective of the edge labels. The argument for a sequence was presented in [69] but is easily generalised to a tree. Given a reversible edge, $i\overset {a}{\rightarrow }j$ and $j\overset {b}{\rightarrow }i$, and a steady state *x*^∗^, the net flux through the reversible edge is $ax^{*}_{i} - bx^{*}_{j}$. If the reversible edge is a leaf of the tree structure then there can be no net flux leaving the tree from that edge. Hence, $x^{*}_{i} = (b/a)x^{*}_{j}$. This reversible edge is therefore at equilibrium. This holds irrespective of the labels *a* and *b*. Arguing in this way by induction from the leaves, each reversible edge in the tree is independently at equilibrium, so that detailed balance holds.

*Rooted spanning trees.* A spanning tree of a graph *G* is a sub-graph that contains each vertex of *G* (spanning) and that has no cycles when edge directions are ignored (tree). A spanning tree is rooted at vertex *j* in *G* if *j* is the only vertex with no outgoing edges. A graph is strongly connected if, and only if, it has at least one rooted spanning tree at each vertex ([37], Lemma 1). Figure 4B shows a strongly connected graph, together with the spanning trees rooted at each vertex.

Terminal strongly connected components. Let [ *j*] denote the SCC of *G* containing vertex *j*. In other words, [ *j*] is the equivalence class of vertex *j* under the relation of strong connectivity, as defined above. The SCC [ *i*] is said to precede [ *j*], denoted [ *i*]≼ [ *j*], if either [ *i*]= [ *j*] or some vertex in [ *i*] ultimately reaches some vertex in [ *j*]: *i*^′^⇝*j*^′^ where *i*^′^∈ [ *i*] and *j*^′^∈ [ *j*]. Precedence defines a partial order on the SCCs of the graph *G*. We can therefore speak of the terminal SCCs, which are those that do not precede any other SCC. The graph in Figure 4C has three SCCs of which two are terminal (asterisks), while the graph in Figure 6C has five SCCs of which two are terminal (asterisks).

### Calculating the *PHO5* gene-regulation function

The gene-regulation function of the *PHO5* example was calculated using the matrix-tree formula in Equation 7 and is shown in full in Additional file 1C. Software for enumerating spanning trees is available in packages like MATLAB, Mathematica and Maple, but we found these to be incapable of dealing with the large number of trees that arise. We therefore implemented in Python the fast algorithm developed by Takeaki Uno [70]. The resulting program reads a text file containing a description of a graph as a collection of labelled edges and, for each vertex in the graph, writes a text file listing the spanning trees rooted at that vertex. We also implemented an accompanying Mathematica notebook, which reads the graph description and the spanning tree files and assembles each ${\rho ^{G}_{i}}$ as a polynomial function of the edge labels. The gene-regulation function can then be calculated using standard Mathematica functions for manipulating polynomial expressions. The Python program and the Mathematica notebook are freely available from our web site [71].

### Fitting to the experimental data of Kim and O’Shea

Kim and O’Shea constructed 12 promoter variants ([52], Figure 3a). Six of these variants place a high affinity (H), low affinity (L) or deleted (X) Pho4-binding site in the positions corresponding to UASp1 and UASp2 in Figure 7A. The remaining six variants use sites occluded by nucleosome -3, which is not modelled in Figure 7, and we did not analyse these variants. The wild-type promoter in Figure 7 corresponds to variant LH.

We obtained the experimental data in the form of an Excel spreadsheet [72]. This gives the raw fluorescence values for YFP, CFP and RFP (yellow, cyan and red fluorescent proteins, respectively) for about 400 to 500 cells for each variant under different doxycycline concentrations. The RFP was attached to a chromatin protein to mark the nucleus and the RFP value was used to normalise the YFP and CFP values on a per-cell basis to control against imaging variations. We used a ±7 moving average to smooth the data and scaled each variant to its maximum expression level for the plots shown in Figure 8.

Each of the six variants gives rise to a graph, which uses the same labels as the wild type (Figure 7B). The labels *b* and *c* are the rates of Pho4 dissociation from the low-affinity and high-affinity sites, respectively. Kim and O’Shea assumed that the Pho4 association rate, *a*, is the same for both sites. If the Pho4 binding sites are changed in a variant, the labels *b* and *c* occur on different edges of the wild-type graph, while if a Pho4 binding site is deleted, some vertices become inaccessible and the graph changes from the 12-vertex wild-type graph to a graph with eight vertices. We used the wild-type 12-vertex gene-regulation function and a new eight-vertex gene-regulation function calculated using Equation 7. We then changed the labels *b* and *c* in these two gene-regulation functions, as required, to generate the gene-regulation function for each of the six variants (details in the accompanying Mathematica notebook).

Kim and O’Shea assumed that the Pho4 association rate, *a*, is a Hill function of Pho4 concentration given by

$$ a = k^{*}_{\text{assoc}} = \frac{k^{*}_{\text{max}}[\!\text{Pho4}]^{2}}{K^{2}\, + \,[\!\text{Pho4}]^{2}}, $$

so that the gene-regulation functions depend on six parameters:

$$ K, k^{*}_{\text{max}}, k^{\text{exp}}_{\text{dissoc}}, k^{\text{nuc}}_{\text{dissoc}}, k_{\text{remod}}\; \text{and}\; k_{\text{reass}}. $$

These have units of concentration, for *K*, and inverse time, for the others. We followed Kim and O’Shea in assuming that [ Pho4]=*α*·nYFP, where nYFP is normalised YFP. The constant of proportionality, *α*, is not known but can be absorbed into the parameter *K*. We therefore left *K* as a dimensional parameter having units of concentration, and used nYFP as the input to the individual gene-regulation functions. We de-dimensionalised the remaining parameters by dividing each by $k^{*}_{\text {max}}$, thereby replacing each edge label *x* by $x/k^{*}_{\text {max}}$, where *x* is one of *a*,*b*,*c*,*d*,*e*, and reducing the number of parameters from six to five. The red curves in Figure 8 were obtained by fitting each variant individually using the Levenberg–Marquardt algorithm in Mathematica. We were unable to do the same for a collective fit because the Levenberg–Marquardt algorithm did not terminate. We therefore used Mathematica to plot the gene-regulation function overlaid against the corresponding smoothed experimental data for each variant and used the Manipulate capability to alter the values of the five parameters manually and to assess the goodness of fit to all the variants visually. We found the following numerical parameter values that yielded the collective fit shown in the black curves in Figure 8,

(16) $$ \begin{aligned} K &= 25,\,\, \frac{k^{\text{exp}}_{\text{dissoc}}}{k^{*}_{\text{max}}} = 0.08,\,\, \frac{k^{\text{nuc}}_{\text{dissoc}}}{k^{*}_{\text{max}}} = 0.02 \\ \frac{k_{\text{remod}}}{k^{*}_{\text{max}}} &= 0.04 \text{ and } \frac{k_{\text{reass}}}{k^{*}_{\text{max}}} = 0.0048.  \end{aligned}  $$

The Mathematica notebook in which these calculations were undertaken is freely available from our web site [71]. It provides the normalised experimental data, the smoothed experimental data and the individual and collective fits of the variant gene-regulation functions to the corresponding data.

### Imposing equilibrium on the Hodges–Crabtree model

As explained in the main text, to impose equilibrium is to require that detailed balance holds. This means, first, that all edges in the graph must be reversible and, second, that the cycle condition (described in the glossary above) is satisfied. The graph of microstates for an array of three nucleosomes is shown in Figure 6B and we follow the notation introduced there in which microstates are denoted by bit strings, indicating whether (bit = 1) or not (bit = 0) a nucleosome is marked. Edges only occur between microstates that differ by a single bit, corresponding to nucleation or mark propagation, when the number of bits increases by 1 and the edge has label *k*+, or to mark turnover, when the number of bits decreases by 1 and the edge has label *k*_ (Figure 6A). Irreversibility only arises for some of the latter edges, when an isolated site, whose immediate neighbours are unmarked, loses its mark (for instance, 5→1, 3→1 and 6→2 in Figure 6B).

To impose reversibility, assume that reverse edges have been introduced into the graph as needed, each with the label *k*+. To check the cycle condition, choose any cycle of reversible edges from a vertex *j* back to itself,

$$ j = i_{1} \rightleftharpoons i_{2} \rightleftharpoons \dots \rightleftharpoons i_{m-1} \rightleftharpoons i_{m} = j. $$

In traversing this path, if an edge increases the number of bits in the microstate by 1, then the label encountered must be *k*+, while if an edge decreases the number of bits by 1, then the label must be *k*_. Since the path is a cycle, the number of edges with label *k*+ must equal the number of edges with label *k*_. Furthermore, for each edge with label *k*+, respectively, *k*_, the reverse edge has label *k*_, respectively, *k*+. But then the product of the labels going clockwise around the cycle must equal the product of the labels going counterclockwise around the cycle and the cycle condition is satisfied. The graph therefore satisfies detailed balance in any steady state.

Equilibrium probabilities can now be calculated using Equation 5. Let *K*=*k*+/*k*_. Given a microstate *j*, let *β*(*j*) be the number of bits in *j* that are set to 1. It is easy to construct a path of reversible edges from the reference microstate 1 to microstate *j* with just *β*(*j*) edges, each of which increases the number of bits by 1. Hence, according to Equation 5,

$$ {\rho^{G}_{j}} = K^{\beta(j)}. $$

If the number of sites in the array is *n*, then the partition function is given by

$$ \sum_{j=1}^{2^{n}} K^{\beta(j)}. $$

However, there are $\binom {n}{\beta (j)}$ microstates each having *β*(*j*) sites marked, so the partition function may be rewritten as

$$ \sum_{i=1}^{n} \binom{n}{i}K^{i} = (1 + K)^{n}. $$

Another way of seeing this is to note that, when equilibrium is imposed, the system becomes identical to *n* independent copies of the one-site system. The partition function can then be calculated from the product formula (Equation 14), which is a special case of the product theorem proved in Additional file 1B. It now follows from Equation 4 that the probability of microstate *j* is given by

$$ \frac{K^{\beta(j)}}{(1 + K)^{n}}. $$

We see from this that the probability of a microstate depends only on the number of bits that are marked, rather than which bits are marked and, consequently, there can be no inherent bound on the size of the marked domain.

## Additional file

Additional file 1
**(A) Calculation of the steroid-hormone gene-regulation function. (B) Proof of the product theorem. (C) Details of the** ***PHO5*** **gene-regulation function.**

## Abbreviations

FHDC: first-order Hill dose–response curve
SCC: strongly connected component
TF: transcription factor
