# Supplementary material for: A framework for modelling gene regulation which accommodates non-equilibrium mechanisms
Source: BMC Biol. 2014 Dec 5;12:102. doi: 10.1186/s12915-014-0102-4 (PMC4288563; doi:10.1186/s12915-014-0102-4)
Supplement: Additional file 1 — (A) Calculation of the steroid-hormone gene-regulation function. (B) Proof of the product theorem. (C) Details of the PHO5 gene-regulation function. [file 12915_2014_102_MOESM1_ESM.pdf]

ADDITIONAL FILE 1

Tobias Ahsendorf[1,3,†] Felix Wong[2,3,†] Roland Eils[1]
Jeremy Gunawardena[3,*]

[1] DKFZ, D-69120 Heidelberg, Germany
[2] Harvard College, Cambridge MA, USA
[3] Department of Systems Biology, Harvard Medical School, Boston MA, USA

[†]Equal contributor
[*]Corresponding author; E-mail: jeremy@hms.harvard.edu

Abstract

This note provides mathematical details for the results presented in the paper *"A framework for modelling gene regulation which accommodates non-equilibrium mechanisms"*. It should be read in conjunction with the paper, which gives further information.

A: Calculation of the steroid hormone gene regulation function

Along with the assumptions made in the Paper, we assume that the microstates of G are enumerated $1, \cdots, n$, and that 1 is the reference microstate in which no components are bound. Recall that components $R, U, Y_1, \cdots, Y_m$ are assumed to be present and that they bind to form the microstates. It will be convenient to denote by X the complex RS of receptor R and steroid hormone S, so that $X = RS$.

Since G is assumed to satisfy detailed balance, a basis element $\rho^G \in \ker \mathcal{L}(G)$ can be calculated using Paper Equation 5. For each microstate i, choose any path of reversible edges from 1 to i,

$$1 = i_1 \underset{b_1}{\overset{a_1}{\rightleftharpoons}} i_2 \underset{b_2}{\overset{a_2}{\rightleftharpoons}} \cdots \underset{b_{p-1}}{\overset{a_{p-1}}{\rightleftharpoons}} i_p \underset{b_p}{\overset{a_p}{\rightleftharpoons}} i_{p+1} = i\,.$$

Binding reactions may give rise to either labels a_i or b_i on this path. If these binding reactions involve the $Y_1, \cdots, Y_m$, then the corresponding labels are constants. If the binding reactions involve R or U, then it is possible for each of these components to bind and unbind as the path above is traversed in the forward direction from 1 to i. If that happens for, say, R, then a binding corresponds to a label of the form $a_i = k[X]$, while an unbinding corresponds to a label of the form $b_j = k'[X]$. Note that k and k' may be different, as these reactions take place in different microstates. However, in forming ρ_i^G by the formula in Paper Equation 5, an occurrence of $[X]$ in the numerator, corresponding to $a_i = k[X]$, will be cancelled by an occurrence of $[X]$ in the denominator, corresponding to $b_j = k'[X]$. Since R and U are assumed to bind at most once in each microstate, we find that

$$\rho_i^G = \alpha_i [X]^{\epsilon_{R,i}} [U]^{\epsilon_{U,i}}\,,$$

where α_i is a constant determined by the rate constants and the total amounts and $\epsilon_{Z,i} = 1, 0$ according as Z is, or is not, bound, respectively, in microstate i. Let us use the notation $Z \in i$ to indicate that the component Z is bound in microstate i. Recall that the partition function of the graph is the sum of ρ_i^G over all microstates, as in the denominator in Paper Equation 4. The partition function can be decomposed as

$$\sum_{1 \leq i \leq n} \rho_i^G = A_0 + A_U[U] + A_R[X] + A_{U,R}[U][X]\,,$$

where A_0, A_U, A_R and $A_{U,R}$ are constants given by

$$\begin{array}{rclrcl} A_0 & = & \sum_{R \notin i, U \notin i} \alpha_i & A_U & = & \sum_{R \notin i, U \in i} \alpha_i \\ A_R & = & \sum_{R \in i, U \notin i} \alpha_i & A_{U,R} & = & \sum_{R \in i, U \in i} \alpha_i\,. \end{array}$$

If U and R could bind more than once in some microstate, then the partition function would be more complicated and would have terms with $[U]^i$ or $[R]^i$ where $i > 1$. The assumption that U and R bind no more than once is therefore particularly significant for what follows.

The probabilities of microstates are obtained by normalising ρ^G to its partition function, according to Paper Equation 4. If the microstate i has both U and R bound, then its steady-state probability of occurrence, considered as a function of $[U]$ and $[X]$, is

$$u_i^*([U], [X]) = \frac{\alpha_i [U][X]}{A_0 + A_U[U] + A_R[X] + A_{U,R}[U][X]}\,.$$

Note that the partition function in the denominator is the same for all i. It follows that if g_i is the rate of gene expression in microstate i and $g_i = 0$ unless both U and R are bound, then the average rate of gene expression is given by

$$\gamma([U],[X]) = \frac{\beta[U][X]}{A_0 + A_U[U] + A_R[X] + A_{U,R}[U][X]}\,, \tag{1}$$

where $\beta = \left(\sum_{U,R\in i} g_i\alpha_i\right) = \left(\sum_{1\leq i\leq n} g_i\alpha_i\right)$.

In [2], Ong *et al* refer to protein amounts, not rates of gene expression, but it is easier to work with the latter and to then briefly explain the assumptions needed to obtain the former.

With the general formula in Eq. 1 to hand, we can now consider U and X. For U, we assume by the non-depletion assumption that $[U] \approx U_{tot}$. As for X, it was assumed to satisfy the FHDC described in the Paper,

$$[X] = \frac{R_{tot}[S]}{K_R + [S]}\,.$$

Here, R_{tot} is the maximal asymptotic value and K_R is the half-maximal concentration. Substituting these expressions for $[U]$ and $[X]$ into Eq. 1 and letting $g([S]) = \gamma(U_{tot}, R_{tot}[S]/(K_R + [S])$, we see that

$$\begin{aligned} g([S]) &= \frac{\beta U_{tot}R_{tot}[S]}{(K_R+[S])(A_0 + A_U U_{tot}) + (A_R + A_{U,R}U_{tot})R_{tot}[S]} \\ &= \frac{\beta U_{tot}R_{tot}[S]}{K_R(A_0 + A_U U_{tot}) + (A_0 + A_U U_{tot} + A_R R_{tot} + A_{U,R}U_{tot}R_{tot})[S]}\,. \end{aligned} \tag{2}$$

The term multiplying $[S]$ in the denominator of Eq. 2 is nothing other than the partition function at saturation (ie: the partition function evaluated when $[U] = U_{tot}$ and when $[X]$ reaches its maximal value of R_{tot}). Dividing above and below by this quantity, $g([S])$ can be rewritten as a FHDC to give Paper Equation 11,

$$g([S]) = \frac{M_G[S]}{K_G + [S]}\,.$$

The parameters of this new FHDC have a natural interpretation. The maximal asymptotic value is the average gene-expression rate at saturation

$$M_G = \gamma(U_{tot}, R_{tot}) \tag{3}$$

and the half-maximal concentration is the original half-maximal concentration, K_R, multiplied by the saturation probability of those microstates in which R is not bound,

$$K_G = K_R\left(\frac{A_0 + A_U U_{tot}}{A_0 + A_U U_{tot} + A_R R_{tot} + A_{U,R}U_{tot}R_{tot}}\right)\,.$$

In [2], Ong *et al* use a different arrangement of Eq. 2, in which the dependence on R_{tot} and U_{tot} is made explicit. They also benefit from a special property of a sequence of edges. In such a sequence, if R binds first, as Ong *et al* assume, then there are no microstates in which U is bound but R is not. Accordingly, $A_U = 0$. We can therefore rewrite Eq. 2 as

$$g([S]) = \frac{(\beta/(K_R A_0))U_{tot}R_{tot}[S]}{1 + ((1/K_R) + (A_R/(K_R A_0))R_{tot} + (A_{U,R}/(K_R A_0))U_{tot}R_{tot})[S]}\,. \tag{4}$$

Ong et al make the further assumption that U exists both as endogenous protein, whose total amount is U^e_{tot}, and as protein introduced into cells by transfection, whose total amount is U^t_{tot}. While U^e_{tot} is unknown, U^t_{tot} can be titrated by experiment. If the equality $U_{tot} = U^e_{tot} + U^t_{tot}$ is substituted into Eq. 4 and U^e_{tot} is absorbed into the parameters, this gives Equation 5 in [2].

Ong *et al* fit the resulting formula to experimental data on the glucocorticoid receptor. The fitted values of the parameters are inconsistent with experimentally measured data, which leads them to introduce a new hypothesis. The assume that some co-regulator Y_j, which is different from either U or R, is at sufficiently low concentration that it no longer satisfies the no-depletion assumption. They describe this as a "concentration limiting step". The effect of such a step is to change the approximate conservation law $[Y_j] \approx Y_{j,tot}$, which we have used here, to the exact law

$$[Y_j] + \left(\sum_{Y_j \in i} u^*_i \right) Y_{j,tot} = Y_{j,tot} \,.$$

Ong *et al* solve the resulting equations for the special case of a sequence and show that, despite the presence of a concentration limiting step, a FHDC still emerges which provides a more consistent fit to the experimental data. We refer to [2] for these details.

Finally, we point out the additional assumptions that are needed to relate the rate of gene expression, as discussed here, with protein amounts, as discussed in [2]. The rate of change of mRNA concentration is a balance between synthesis (ie: expression, as calculated here) and degradation. If mRNA is assumed to be linearly degraded and if protein concentration at steady state is assumed to depend linearly on mRNA concentration, then the steady-state protein concentration is just a multiple of the gene expression rate given by $g([S])$. Hence, any discussion of FHDCs applies equally well in either interpretation, the only difference being that the maximal protein amount is proportional to the formula in Eq. 3.

B: Proof of the Product Theorem

Let G and H be two labelled, directed graphs and suppose that G has n vertices, $1, \cdots, n$, and H has m vertices, $1, \cdots, m$. Recall from the Paper that the product graph $G \times H$ is constructed as follows. It has vertices (i, j) where i is a vertex of G and j is a vertex of H. If $i_1 \xrightarrow{a} i_2$ is an edge in G, then there is an edge $(i_1, j) \xrightarrow{a} (i_2, j)$ in $G \times H$, for every vertex j in H. Similarly, if $j_1 \xrightarrow{b} j_2$ is an edge in H, then there is an edge $(i, j_1) \xrightarrow{b} (i, j_2)$ in $G \times H$ for every vertex i in G. These are the only edges in $G \times H$.

We impose the lexicographic (dictionary) ordering on the vertices of $G \times H$, so that $(i, j) < (i', j')$ if either $i < i'$ or $i = i'$ and $j < j'$, and we use this ordering when constructing the Laplacian matrix of $G \times H$. The vertex (i, j) is then the $((i-1)m + j)$-th vertex in the ordering.

Suppose now that the Laplacian kernels of G and H have bases given by

$$\ker \mathcal{L}(G) = \langle \rho^{G,1}, \cdots, \rho^{G,s} \rangle \quad \text{and} \quad \ker \mathcal{L}(H) = \langle \rho^{H,1}, \cdots, \rho^{H,t} \rangle \,. \tag{5}$$

The bases constructed as described in Paper Equation 8 have the characteristic property that the *supports* of the basis vectors are in one-to-one correspondence with the terminal strongly connected components of the corresponding graphs. If v is a column vector, its support, denoted supp(v), is the set of indices on which v is non-zero: supp$(v) = \{i \mid v_i \neq 0\}$. By following the construction in the Paper, the terminal

strongly connected components of G and H can be enumerated, $T^{G,1}, \cdots, T^{G,s}$ and $T^{H,1}, \cdots, T^{H,t}$ in such a way that

$$\text{supp}(\rho^{G,k}) = T^{G,k} \quad \text{and} \quad \text{supp}(\rho^{H,l}) = T^{H,l} \,. \tag{6}$$

We will say that a basis for the kernel of a Laplacian matrix is *aligned* if there is such a one-to-one correspondence between the supports of the basis vectors and the terminal strongly connected components of the underlying graph. Note that if there are two or more strongly connected components, it is easy to construct a basis which is not aligned.

The **Product Theorem** states that, given aligned bases for the Laplacian kernels of G and H, as in Eq. 5, then the Laplacian kernel of $G \times H$ has an aligned basis of Kronecker products,

$$\ker \mathcal{L}(G \times H) = \langle \rho^{G,1} \otimes \rho^{H,1}, \cdots, \rho^{G,k} \otimes \rho^{H,l}, \cdots, \rho^{G,s} \otimes \rho^{H,t} \rangle \,. \tag{7}$$

This result generalises to dissipative, non-equilibrium systems the product formula for partition functions in Paper Equation 13, which is widely used in the equilibrium thermodynamic formalism.

The Kronecker product is defined for matrices as follows (the Kronecker product for vectors follows by specialisation.) Suppose that A is a $p \times q$ matrix and B is a $u \times v$ matrix. The Kronecker product, $A \otimes B$, is the $pu \times qv$ matrix given by

$$A \otimes B = \begin{pmatrix} A_{1,1}B & \cdots & A_{1,q}B \\ \vdots & \ddots & \vdots \\ A_{p,1}B & \cdots & A_{p,q}B \end{pmatrix} .$$

Alternatively, $A \otimes B$ can be defined by its entries: if $1 \leq k \leq pu$ and $1 \leq l \leq qv$, then

$$(A \otimes B)_{k,l} = A_{\lceil \frac{k}{u} \rceil, \lceil \frac{l}{v} \rceil} B_{k \bmod u, l \bmod v} \,. \tag{8}$$

Here, $\lceil x \rceil$ denotes the ceiling function, or the smallest integer which is not strictly smaller than x. For k and u integers, $k \bmod u$ denotes the remainder obtained after dividing k by u, with the proviso that, if u divides k, so that the remainder would normally be zero, then $k \bmod u = u$.

The Kronecker product satisfies the following useful identity. If C is a $q \times r$ matrix and D is a $v \times w$ matrix, then

$$(A \otimes B).(C \otimes D) = A.C \otimes B.D \,, \tag{9}$$

which holds because

$$(A \otimes B).(C \otimes D) = \begin{pmatrix} A_{1,1}B & \cdots & A_{1,q}B \\ \vdots & \ddots & \vdots \\ A_{p,1}B & \cdots & A_{p,q}B \end{pmatrix} . \begin{pmatrix} C_{1,1}D & \cdots & C_{1,r}D \\ \vdots & \ddots & \vdots \\ C_{q,1}D & \cdots & C_{q,r}D \end{pmatrix} =$$

$$\begin{pmatrix} \left(\sum_{i=1}^{q} A_{1,i}C_{i,1}\right) B.D & \cdots & \left(\sum_{i=1}^{q} A_{1,i}C_{i,r}\right) B.D \\ \vdots & \ddots & \vdots \\ \left(\sum_{i=1}^{q} A_{p,i}C_{i,1}\right) B.D & \cdots & \left(\sum_{i=1}^{q} A_{p,i}C_{i,r}\right) B.D \end{pmatrix} = A.C \otimes B.D$$

With this preparation, we can check that

$$\mathcal{L}(G \times H) = \mathcal{L}(G) \otimes I_m + I_n \otimes \mathcal{L}(H) \,, \tag{10}$$

where I_k denotes the $k \times k$ identity matrix. To see this, let us focus on an off-diagonal element of $\mathcal{L}(G \times H)$, which corresponds to an edge $(i,j) \xleftarrow{a} (i',j')$ in $G \times H$. By construction of $G \times H$, such an edge only occurs if $i \xleftarrow{a} i'$ in G and $j = j'$ or $j \xleftarrow{a} j'$ in H and $i = i'$. Suppose the former, so that

$$a = \mathcal{L}(G \times H)_{(i,j),(i',j)} = \mathcal{L}(G \times H)_{(i-1)m+j,(i'-1)m+j}\,,$$

and compare this with the two Kronecker product summands on the right of Eq. 10. According to Eq. 8,

$$(\mathcal{L}(G) \otimes I_m)_{(i-1)m+j,(i'-1)m+j} =$$
$$\mathcal{L}(G)_{\lceil \frac{(i-1)m+j}{m} \rceil, \lceil \frac{(i'-1)m+j}{m} \rceil} (I_m)_{((i-1)m+j) \bmod m, ((i'-1)m+j) \bmod m} =$$
$$\mathcal{L}(G)_{i,i'}(I_m)_{j,j} = a\,,$$

while, for the other summand, noting that $i \neq i'$,

$$(I_n \otimes \mathcal{L}(H))_{(i-1)m+j,(i'-1)m+j} = (I_n)_{i,i'}\mathcal{L}(H)_{j,j} = 0\,,$$

In a similar way, the second summand $I_n \otimes \mathcal{L}(H)$ accounts for those edges in $G \times H$ which come from edges in H, with the first summand contributing 0. It follows that Eq. 10 holds for the off-diagonal elements of the matrices. As for the diagonal elements, these are determined by the off-diagonal elements through the requirement that the column sums of each matrix in Eq. 10 are zero. For the Laplacian matrix on the left, we already know that $1.\mathcal{L}(G \times H) = 0$. For the summands on the right, we can write the all-one's row vector as a Kronecker product, $1_{mn} = 1_n \otimes 1_m$, so that, using Eq. 9,

$$1_{mn}.(\mathcal{L}(G) \otimes I_m) = (1_n \otimes 1_m).(\mathcal{L}(G) \otimes I_m) = 1_n.\mathcal{L}(G) \otimes 1_m.I_m = 0 \otimes 1_m = 0\,,$$

and, similarly, $1_{mn}.(I_n \otimes \mathcal{L}(H)) = 0$. Eq. 10 follows.

Eq. 10 makes it easy to see that the Kronecker product vectors in Eq. 7 are in the kernel of the Laplacian matrix of $G \times H$. Using Eq. 9 as well, we see that

$$\begin{aligned}\mathcal{L}(G \times H).(\rho^{G,k} \otimes \rho^{H,l}) &= (\mathcal{L}(G) \otimes I_m).(\rho^{G,k} \otimes \rho^{H,l}) + (I_n \otimes \mathcal{L}(H)).(\rho^{G,k} \otimes \rho^{H,l}) \\ &= \mathcal{L}(G).\rho^{G,k} \otimes I_m.\rho^{H,l} + I_n.\rho^{G,k} \otimes \mathcal{L}(H).\rho^{H,l} = 0 \otimes \rho^{H,l} + \rho^{G,k} \otimes 0 = 0\,.\end{aligned}$$

It follows that $\rho^{G,k} \otimes \rho^{H,l} \in \ker \mathcal{L}(G \times H)$ for $1 \leq k \leq s$ and $1 \leq l \leq t$.

To finish the proof, we need to show that these Kronecker products form an aligned basis for the kernel of $\mathcal{L}(G \times H)$. We first compute their supports. Each Kronecker product $\rho^{G,k} \otimes \rho^{H,l}$ is a column vector of size nm. If (i,j) is a vertex in $G \times H$, so that $1 \leq i \leq n$ and $1 \leq j \leq m$, then using Eq. 8, we see that the component of the Kronecker product at this vertex is

$$\begin{gathered}(\rho^{G,k} \otimes \rho^{H,l})_{(i,j)} = (\rho^{G,k} \otimes \rho^{H,l})_{(i-1)m+j} = \\ (\rho^{G,k})_{\lceil \frac{(i-1)m+j}{m} \rceil} (\rho^{H,l})_{((i-1)m+j) \bmod m} = (\rho^{G,k})_i (\rho^{H,l})_j\,.\end{gathered} \tag{11}$$

(Note that this was how the Kronecker product of two vectors was defined in Paper Equation 14.) If A and B are subsets of vertices in G and H, respectively, $A \subseteq \{1, \cdots, n\}$ and $B \subseteq \{1, \cdots, m\}$, let $A \times B$ denote the product subset in $G \times H$: $A \times B = \{(i,j) \,|\, i \in A, j \in B\}$. It follows easily from Eq. 11 that

$$\text{supp}(\rho^{G,k} \otimes \rho^{H,l}) = \text{supp}(\rho^{G,k}) \times \text{supp}(\rho^{H,l})$$

so that, using Eq. 6,

$$\mathrm{supp}(\rho^{G,k} \otimes \rho^{H,l}) = T^{G,k} \times T^{H,l} \,. \tag{12}$$

The proof would be finished if we can show that the supports in Eq. 12 correspond to the terminal strongly connected components of $G \times H$. This would establish that the Kronecker product vectors are linearly independent, since their supports are pairwise mutually disjoint, and that there are sufficiently many such vectors to make a basis for $\ker \mathcal{L}(G \times H)$, since $\dim \ker \mathcal{L}(G \times H) = st$, which is the number of terminal strongly connected components, and, finally, that this basis is aligned. Recall the notation introduced in the Materials and Methods of the paper. Suppose that $i \rightsquigarrow i'$ in G and $j \rightsquigarrow j'$ in H, then it follows from the construction of $G \times H$ that $(i, j) \rightsquigarrow (i', j) \rightsquigarrow (i', j')$ in $G \times H$. Conversely, if $(i, j) \rightsquigarrow (i', j')$ in $G \times H$, then, by projecting onto G and onto H, it is easy to see that $i \rightsquigarrow i'$ in G and $j \rightsquigarrow j'$ in H, respectively. It follows that $(i, j) \rightsquigarrow (i', j')$ in $G \times H$, if, and only if, $i \rightsquigarrow i'$ in G and $j \rightsquigarrow j'$ in H. It is then easy to check that the strongly connected components of $G \times H$ are precisely the products of the strongly connected components of G and H: $[(i, j)] = [i] \times [j]$. Furthermore, the partial order on strongly connected components in $G \times H$ corresponds through this mapping to the product partial order: $[(i, j)] \preceq [(i', j')]$ in $G \times H$ if, and only if, $[i] \preceq [i']$ in G and $[j] \preceq [j']$ in H. It follows that the terminal strongly connected components of $G \times H$ are precisely the products of the terminal strongly connected components of G and H, which correspond to the supports of the Kronecker product vectors in Eq. 12. Hence,

$$\{\rho^{G,1} \otimes \rho^{H,1}, \cdots, \rho^{G,k} \otimes \rho^{H,l}, \cdots, \rho^{G,s} \otimes \rho^{H,t}\} \,.$$

is an aligned basis for $\ker \mathcal{L}(G \times H)$. The Product Theorem follows.

C: Details of the *PHO5* gene regulation function

The twelve components of the basis vector $\rho^G \in \ker \mathcal{L}(G)$ are listed below for the *PHO5* example, following the notation introduced in Paper Figure 7B. The gene regulation function calculated in the original paper, [1], is given by the fraction

$$\frac{\rho_2^G + \rho_3^G + \rho_7^G + \rho_8^G + \rho_9^G + \rho_{12}^G}{\sum_{i=1}^{12} \rho_i^G} \,,$$

where the numerator has those microstates, $2, 3, 7, 8, 9, 12$, in which the TATA box is not obscured by a nucleosome and in which transcription is presumed to occur.

$$\rho_1^G(a, b, c, d, e) =$$

$4a^7bd^2e+8a^6b^2d^2e+5a^5b^3d^2e+a^4b^4d^2e+8a^6bcd^2e+14a^5b^2cd^2e+7a^4b^3cd^2e+a^3b^4cd^2e+5a^5bc^2d^2e+ 7a^4b^2c^2d^2e + 2a^3b^3c^2d^2e + a^4bc^3d^2e + a^3b^2c^3d^2e + 8a^6bd^3e + 10a^5b^2d^3e + 3a^4b^3d^3e + 12a^5bcd^3e + 11a^4b^2cd^3e + 2a^3b^3cd^3e + 6a^4bc^2d^3e + 3a^3b^2c^2d^3e + a^3bc^3d^3e + 4a^5bd^4e + 2a^4b^2d^4e + 4a^4bcd^4e + a^3b^2cd^4e + a^3bc^2d^4e + 4a^7bde^2 + 12a^6b^2de^2 + 13a^5b^3de^2 + 6a^4b^4de^2 + a^3b^5de^2 + 12a^6bcde^2 + 34a^5b^2cde^2 + 34a^4b^3cde^2 + 14a^3b^4cde^2 + 2a^2b^5cde^2 + 9a^5bc^2de^2 + 22a^4b^2c^2de^2 + 17a^3b^3c^2de^2 + 4a^2b^4c^2de^2 + 2a^4bc^3de^2 + 4a^3b^2c^3de^2 + 2a^2b^3c^3de^2 + 26a^6bd^2e^2 + 47a^5b^2d^2e^2 + 27a^4b^3d^2e^2 + 5a^3b^4d^2e^2 + 45a^5bcd^2e^2 + 71a^4b^2cd^2e^2 + 34a^3b^3cd^2e^2 + 5a^2b^4cd^2e^2 + 24a^4bc^2d^2e^2 + 29a^3b^2c^2d^2e^2 + 8a^2b^3c^2d^2e^2 + 4a^3bc^3d^2e^2 + 3a^2b^2c^3d^2e^2 + 32a^5bd^3e^2 + 33a^4b^2d^3e^2 + 8a^3b^3d^3e^2 + 37a^4bcd^3e^2 +$

$26a^3b^2cd^3e^2 + 4a^2b^3cd^3e^2 + 12a^3bc^2d^3e^2 + 5a^2b^2c^2d^3e^2 + a^2bc^3d^3e^2 + 10a^4bd^4e^2 + 4a^3b^2d^4e^2 +$
$6a^3bcd^4e^2 + a^2b^2cd^4e^2 + a^2bc^2d^4e^2 + 22a^6bde^3 + 55a^5b^2de^3 + 48a^4b^3de^3 + 17a^3b^4de^3 + 2a^2b^5de^3 +$
$49a^5bcde^3 + 103a^4b^2cde^3 + 68a^3b^3cde^3 + 14a^2b^4cde^3 + 31a^4bc^2de^3 + 49a^3b^2c^2de^3 + 18a^2b^3c^2de^3 +$
$6a^3bc^3de^3+6a^2b^2c^3de^3+64a^5bd^2e^3+96a^4b^2d^2e^3+44a^3b^3d^2e^3+6a^2b^4d^2e^3+84a^4bcd^2e^3+94a^3b^2cd^2e^3+$
$24a^2b^3cd^2e^3 + 32a^3bc^2d^2e^3 + 21a^2b^2c^2d^2e^3 + 3a^2bc^3d^2e^3 + 46a^4bd^3e^3 + 36a^3b^2d^3e^3 + 6a^2b^3d^3e^3 +$
$35a^3bcd^3e^3+12a^2b^2cd^3e^3+6a^2bc^2d^3e^3+8a^3bd^4e^3+2a^2b^2d^4e^3+2a^2bcd^4e^3+46a^5bde^4+88a^4b^2de^4+$
$53a^3b^3de^4 + 10a^2b^4de^4 + 75a^4bcde^4 + 104a^3b^2cde^4 + 34a^2b^3cde^4 + 34a^3bc^2de^4 + 26a^2b^2c^2de^4 +$
$4a^2bc^3de^4 + 74a^4bd^2e^4 + 79a^3b^2d^2e^4 + 20a^2b^3d^2e^4 + 63a^3bcd^2e^4 + 35a^2b^2cd^2e^4 + 13a^2bc^2d^2e^4 +$
$28a^3bd^3e^4+12a^2b^2d^3e^4+10a^2bcd^3e^4+2a^2bd^4e^4+46a^4bde^5+59a^3b^2de^5+18a^2b^3de^5+50a^3bcde^5+$
$34a^2b^2cde^5+12a^2bc^2de^5+40a^3bd^2e^5+22a^2b^2d^2e^5+16a^2bcd^2e^5+6a^2bd^3e^5+22a^3bde^6+14a^2b^2de^6+$
$12a^2bcde^6 + 8a^2bd^2e^6 + 4a^2bde^7$

$$\rho_2^G(a,b,c,d,e) =$$

$4a^7bd^3 + 8a^6b^2d^3 + 5a^5b^3d^3 + a^4b^4d^3 + 8a^6bcd^3 + 10a^5b^2cd^3 + 3a^4b^3cd^3 + 5a^5bc^2d^3 + 3a^4b^2c^2d^3 +$
$a^4bc^3d^3 + 8a^6bd^4 + 10a^5b^2d^4 + 3a^4b^3d^4 + 10a^5bcd^4 + 6a^4b^2cd^4 + 3a^4bc^2d^4 + 4a^5bd^5 + 2a^4b^2d^5 +$
$2a^4bcd^5 + 4a^7bd^2e + 12a^6b^2d^2e + 13a^5b^3d^2e + 6a^4b^4d^2e + a^3b^5d^2e + 8a^6bcd^2e + 18a^5b^2cd^2e +$
$13a^4b^3cd^2e+3a^3b^4cd^2e+5a^5bc^2d^2e+8a^4b^2c^2d^2e+3a^3b^3c^2d^2e+a^4bc^3d^2e+a^3b^2c^3d^2e+26a^6bd^3e+$
$47a^5b^2d^3e+27a^4b^3d^3e+5a^3b^4d^3e+39a^5bcd^3e+44a^4b^2cd^3e+12a^3b^3cd^3e+17a^4bc^2d^3e+9a^3b^2c^2d^3e+$
$2a^3bc^3d^3e + 32a^5bd^4e + 33a^4b^2d^4e + 8a^3b^3d^4e + 29a^4bcd^4e + 14a^3b^2cd^4e + 6a^3bc^2d^4e + 10a^4bd^5e +$
$4a^3b^2d^5e+4a^3bcd^5e+22a^6bd^2e^2+55a^5b^2d^2e^2+48a^4b^3d^2e^2+17a^3b^4d^2e^2+2a^2b^5d^2e^2+35a^5bcd^2e^2+$
$62a^4b^2cd^2e^2+33a^3b^3cd^2e^2+5a^2b^4cd^2e^2+16a^4bc^2d^2e^2+18a^3b^2c^2d^2e^2+4a^2b^3c^2d^2e^2+2a^3bc^3d^2e^2+$
$a^2b^2c^3d^2e^2 + 64a^5bd^3e^2 + 96a^4b^2d^3e^2 + 44a^3b^3d^3e^2 + 6a^2b^4d^3e^2 + 69a^4bcd^3e^2 + 63a^3b^2cd^3e^2 +$
$12a^2b^3cd^3e^2 + 20a^3bc^2d^3e^2 + 7a^2b^2c^2d^3e^2 + a^2bc^3d^3e^2 + 46a^4bd^4e^2 + 36a^3b^2d^4e^2 + 6a^2b^3d^4e^2 +$
$29a^3bcd^4e^2+9a^2b^2cd^4e^2+3a^2bc^2d^4e^2+8a^3bd^5e^2+2a^2b^2d^5e^2+2a^2bcd^5e^2+46a^5bd^2e^3+88a^4b^2d^2e^3+$
$53a^3b^3d^2e^3+10a^2b^4d^2e^3+58a^4bcd^2e^3+73a^3b^2cd^2e^3+22a^2b^3cd^2e^3+18a^3bc^2d^2e^3+11a^2b^2c^2d^2e^3+$
$a^2bc^3d^2e^3 + 74a^4bd^3e^3 + 79a^3b^2d^3e^3 + 20a^2b^3d^3e^3 + 54a^3bcd^3e^3 + 30a^2b^2cd^3e^3 + 8a^2bc^2d^3e^3 +$
$28a^3bd^4e^3+12a^2b^2d^4e^3+10a^2bcd^4e^3+2a^2bd^5e^3+46a^4bd^2e^4+59a^3b^2d^2e^4+18a^2b^3d^2e^4+43a^3bcd^2e^4+$
$29a^2b^2cd^2e^4+7a^2bc^2d^2e^4+40a^3bd^3e^4+22a^2b^2d^3e^4+16a^2bcd^3e^4+6a^2bd^4e^4+22a^3bd^2e^5+14a^2b^2d^2e^5+$
$12a^2bcd^2e^5 + 8a^2bd^3e^5 + 4a^2bd^2e^6$

$$\rho_3^G(a,b,c,d,e) =$$

$4a^8d^3+8a^7bd^3+5a^6b^2d^3+a^5b^3d^3+8a^7cd^3+10a^6bcd^3+3a^5b^2cd^3+5a^6c^2d^3+3a^5bc^2d^3+a^5c^3d^3+$
$8a^7d^4 + 10a^6bd^4 + 3a^5b^2d^4 + 10a^6cd^4 + 6a^5bcd^4 + 3a^5c^2d^4 + 4a^6d^5 + 2a^5bd^5 + 2a^5cd^5 + 4a^8d^2e +$
$12a^7bd^2e + 13a^6b^2d^2e + 6a^5b^3d^2e + a^4b^4d^2e + 8a^7cd^2e + 18a^6bcd^2e + 13a^5b^2cd^2e + 3a^4b^3cd^2e +$
$5a^6c^2d^2e + 8a^5bc^2d^2e + 3a^4b^2c^2d^2e + a^5c^3d^2e + a^4bc^3d^2e + 26a^7d^3e + 47a^6bd^3e + 27a^5b^2d^3e +$
$5a^4b^3d^3e + 43a^6cd^3e + 48a^5bcd^3e + 13a^4b^2cd^3e + 23a^5c^2d^3e + 12a^4bc^2d^3e + 4a^4c^3d^3e + 32a^6d^4e +$
$33a^5bd^4e+8a^4b^2d^4e+35a^5cd^4e+18a^4bcd^4e+10a^4c^2d^4e+10a^5d^5e+4a^4bd^5e+6a^4cd^5e+22a^7d^2e^2+$
$55a^6bd^2e^2 + 48a^5b^2d^2e^2 + 17a^4b^3d^2e^2 + 2a^3b^4d^2e^2 + 37a^6cd^2e^2 + 65a^5bcd^2e^2 + 34a^4b^2cd^2e^2 +$
$5a^3b^3cd^2e^2 + 21a^5c^2d^2e^2 + 25a^4bc^2d^2e^2 + 6a^3b^2c^2d^2e^2 + 4a^4c^3d^2e^2 + 3a^3bc^3d^2e^2 + 64a^6d^3e^2 +$
$96a^5bd^3e^2 + 44a^4b^2d^3e^2 + 6a^3b^3d^3e^2 + 87a^5cd^3e^2 + 80a^4bcd^3e^2 + 16a^3b^2cd^3e^2 + 38a^4c^2d^3e^2 +$
$16a^3bc^2d^3e^2+5a^3c^3d^3e^2+46a^5d^4e^2+36a^4bd^4e^2+6a^3b^2d^4e^2+45a^4cd^4e^2+18a^3bcd^4e^2+11a^3c^2d^4e^2+$
$8a^4d^5e^2 + 2a^3bd^5e^2 + 6a^3cd^5e^2 + 46a^6d^2e^3 + 88a^5bd^2e^3 + 53a^4b^2d^2e^3 + 10a^3b^3d^2e^3 + 67a^5cd^2e^3 +$
$85a^4bcd^2e^3 + 26a^3b^2cd^2e^3 + 32a^4c^2d^2e^3 + 26a^3bc^2d^2e^3 + 4a^2b^2c^2d^2e^3 + 5a^3c^3d^2e^3 + 2a^2bc^3d^2e^3 +$

$74a^5d^3e^3+79a^4bd^3e^3+20a^3b^2d^3e^3+84a^4cd^3e^3+55a^3bcd^3e^3+4a^2b^2cd^3e^3+28a^3c^2d^3e^3+8a^2bc^2d^3e^3+2a^2c^3d^3e^3+28a^4d^4e^3+12a^3bd^4e^3+26a^3cd^4e^3+6a^2bcd^4e^3+4a^2c^2d^4e^3+2a^3d^5e^3+2a^2cd^5e^3+46a^5d^2e^4+59a^4bd^2e^4+18a^3b^2d^2e^4+58a^4cd^2e^4+45a^3bcd^2e^4+4a^2b^2cd^2e^4+22a^3c^2d^2e^4+10a^2bc^2d^2e^4+2a^2c^3d^2e^4+40a^4d^3e^4+22a^3bd^3e^4+40a^3cd^3e^4+14a^2bcd^3e^4+8a^2c^2d^3e^4+6a^3d^4e^4+6a^2cd^4e^4+22a^4d^2e^5+14a^3bd^2e^5+24a^3cd^2e^5+8a^2bcd^2e^5+6a^2c^2d^2e^5+8a^3d^3e^5+8a^2cd^3e^5+4a^3d^2e^6+4a^2cd^2e^6$

$$\rho_4^G(a,b,c,d,e) =$$

$4a^8d^2e+8a^7bd^2e+5a^6b^2d^2e+a^5b^3d^2e+8a^7cd^2e+14a^6bcd^2e+7a^5b^2cd^2e+a^4b^3cd^2e+5a^6c^2d^2e+7a^5bc^2d^2e+2a^4b^2c^2d^2e+a^5c^3d^2e+a^4bc^3d^2e+8a^7d^3e+10a^6bd^3e+3a^5b^2d^3e+10a^6cd^3e+10a^5bcd^3e+2a^4b^2cd^3e+3a^5c^2d^3e+2a^4bc^2d^3e+4a^6d^4e+2a^5bd^4e+2a^5cd^4e+4a^8de^2+12a^7bde^2+13a^6b^2de^2+6a^5b^3de^2+a^4b^4de^2+12a^7cde^2+34a^6bcde^2+34a^5b^2cde^2+14a^4b^3cde^2+2a^3b^4cde^2+9a^6c^2de^2+22a^5bc^2de^2+17a^4b^2c^2de^2+4a^3b^3c^2de^2+2a^5c^3de^2+4a^4bc^3de^2+2a^3b^2c^3de^2+26a^7d^2e^2+47a^6bd^2e^2+27a^5b^2d^2e^2+5a^4b^3d^2e^2+47a^6cd^2e^2+76a^5bcd^2e^2+38a^4b^2cd^2e^2+6a^3b^3cd^2e^2+25a^5c^2d^2e^2+31a^4bc^2d^2e^2+9a^3b^2c^2d^2e^2+4a^4c^3d^2e^2+3a^3bc^3d^2e^2+32a^6d^3e^2+33a^5bd^3e^2+8a^4b^2d^3e^2+35a^5cd^3e^2+28a^4bcd^3e^2+5a^3b^2cd^3e^2+10a^4c^2d^3e^2+5a^3bc^2d^3e^2+10a^5d^4e^2+4a^4bd^4e^2+6a^4cd^4e^2+22a^7de^3+55a^6bde^3+48a^5b^2de^3+17a^4b^3de^3+2a^3b^4de^3+51a^6cde^3+108a^5bcde^3+72a^4b^2cde^3+15a^3b^3cde^3+36a^5c^2de^3+61a^4bc^2de^3+27a^3b^2c^2de^3+2a^2b^3c^2de^3+8a^4c^3de^3+10a^3bc^3de^3+2a^2b^2c^3de^3+64a^6d^2e^3+96a^5bd^2e^3+44a^4b^2d^2e^3+6a^3b^3d^2e^3+97a^5cd^2e^3+118a^4bcd^2e^3+37a^3b^2cd^2e^3+2a^2b^3cd^2e^3+44a^4c^2d^2e^3+37a^3bc^2d^2e^3+4a^2b^2c^2d^2e^3+5a^3c^3d^2e^3+2a^2bc^3d^2e^3+46a^5d^3e^3+36a^4bd^3e^3+6a^3b^2d^3e^3+45a^4cd^3e^3+22a^3bcd^3e^3+2a^2b^2cd^3e^3+11a^3c^2d^3e^3+2a^2bc^2d^3e^3+8a^4d^4e^3+2a^3bd^4e^3+6a^3cd^4e^3+46a^6de^4+88a^5bde^4+53a^4b^2de^4+10a^3b^3de^4+85a^5cde^4+124a^4bcde^4+46a^3b^2cde^4+2a^2b^3cde^4+51a^4c^2de^4+53a^3bc^2de^4+10a^2b^2c^2de^4+10a^3c^3de^4+6a^2bc^3de^4+74a^5d^2e^4+79a^4bd^2e^4+20a^3b^2d^2e^4+92a^4cd^2e^4+71a^3bcd^2e^4+10a^2b^2cd^2e^4+34a^3c^2d^2e^4+14a^2bc^2d^2e^4+2a^2c^3d^2e^4+28a^4d^3e^4+12a^3bd^3e^4+26a^3cd^3e^4+6a^2bcd^3e^4+4a^2c^2d^3e^4+2a^3d^4e^4+2a^2cd^4e^4+46a^5de^5+59a^4bde^5+18a^3b^2de^5+68a^4cde^5+59a^3bcde^5+8a^2b^2cde^5+32a^3c^2de^5+16a^2bc^2de^5+4a^2c^3de^5+40a^4d^2e^5+22a^3bd^2e^5+42a^3cd^2e^5+16a^2bcd^2e^5+10a^2c^2d^2e^5+6a^3d^3e^5+6a^2cd^3e^5+22a^4de^6+14a^3bde^6+26a^3cde^6+10a^2bcde^6+8a^2c^2de^6+8a^3d^2e^6+8a^2cd^2e^6+4a^3de^7+4a^2cde^7$

$$\rho_5^G(a,b,c,d,e) =$$

$4a^7cd^2e+8a^6bcd^2e+5a^5b^2cd^2e+a^4b^3cd^2e+8a^6c^2d^2e+14a^5bc^2d^2e+7a^4b^2c^2d^2e+a^3b^3c^2d^2e+5a^5c^3d^2e+7a^4bc^3d^2e+2a^3b^2c^3d^2e+a^4c^4d^2e+a^3bc^4d^2e+8a^6cd^3e+12a^5bcd^3e+6a^4b^2cd^3e+a^3b^3cd^3e+10a^5c^2d^3e+11a^4bc^2d^3e+3a^3b^2c^2d^3e+3a^4c^3d^3e+2a^3bc^3d^3e+4a^5cd^4e+4a^4bcd^4e+a^3b^2cd^4e+2a^4c^2d^4e+a^3bc^2d^4e+4a^7cde^2+12a^6bcde^2+13a^5b^2cde^2+6a^4b^3cde^2+a^3b^4cde^2+12a^6c^2de^2+34a^5bc^2de^2+34a^4b^2c^2de^2+14a^3b^3c^2de^2+2a^2b^4c^2de^2+9a^5c^3de^2+22a^4bc^3de^2+17a^3b^2c^3de^2+4a^2b^3c^3de^2+2a^4c^4de^2+4a^3bc^4de^2+2a^2b^2c^4de^2+30a^6cd^2e^2+65a^5bcd^2e^2+53a^4b^2cd^2e^2+20a^3b^3cd^2e^2+3a^2b^4cd^2e^2+51a^5c^2d^2e^2+91a^4bc^2d^2e^2+54a^3b^2c^2d^2e^2+11a^2b^3c^2d^2e^2+26a^4c^3d^2e^2+34a^3bc^3d^2e^2+11a^2b^2c^3d^2e^2+4a^3c^4d^2e^2+3a^2bc^4d^2e^2+36a^5cd^3e^2+59a^4bcd^3e^2+35a^3b^2cd^3e^2+7a^2b^3cd^3e^2+37a^4c^2d^3e^2+45a^3bc^2d^3e^2+14a^2b^2c^2d^3e^2+10a^3c^3d^3e^2+7a^2bc^3d^3e^2+10a^4cd^4e^2+14a^3bcd^4e^2+4a^2b^2cd^4e^2+6a^3c^2d^4e^2+4a^2bc^2d^4e^2+22a^6cde^3+57a^5bcde^3+53a^4b^2cde^3+21a^3b^3cde^3+3a^2b^4cde^3+51a^5c^2de^3+113a^4bc^2de^3+84a^3b^2c^2de^3+24a^2b^3c^2de^3+2ab^4c^2de^3+36a^4c^3de^3+63a^3bc^3de^3+31a^2b^2c^3de^3+4ab^3c^3de^3+8a^3c^4de^3+10a^2bc^4de^3+2ab^2c^4de^3+78a^5cd^2e^3+152a^4bcd^2e^3+106a^3b^2cd^2e^3+29a^2b^3cd^2e^3+2ab^4cd^2e^3+112a^4c^2d^2e^3+166a^3bc^2d^2e^3+74a^2b^2c^2d^2e^3+8ab^3c^2d^2e^3+$

$48a^3c^3d^2e^3 + 46a^2bc^3d^2e^3 + 8ab^2c^3d^2e^3 + 5a^2c^4d^2e^3 + 2abc^4d^2e^3 + 56a^4cd^3e^3 + 84a^3bcd^3e^3 + 37a^2b^2cd^3e^3 + 4ab^3cd^3e^3 + 51a^3c^2d^3e^3 + 48a^2bc^2d^3e^3 + 8ab^2c^2d^3e^3 + 11a^2c^3d^3e^3 + 4abc^3d^3e^3 + 8a^3cd^4e^3 + 10a^2bcd^4e^3 + 2ab^2cd^4e^3 + 6a^2c^2d^4e^3 + 2abc^2d^4e^3 + 46a^5cde^4 + 98a^4bcde^4 + 73a^3b^2cde^4 + 22a^2b^3cde^4 + 2ab^4cde^4 + 85a^4c^2de^4 + 141a^3bc^2de^4 + 73a^2b^2c^2de^4 + 12ab^3c^2de^4 + 51a^3c^3de^4 + 59a^2bc^3de^4 + 16ab^2c^3de^4 + 10a^2c^4de^4 + 6abc^4de^4 + 92a^4cd^2e^4 + 147a^3bcd^2e^4 + 76a^2b^2cd^2e^4 + 12ab^3cd^2e^4 + 111a^3c^2d^2e^4 + 119a^2bc^2d^2e^4 + 30ab^2c^2d^2e^4 + 39a^2c^3d^2e^4 + 20abc^3d^2e^4 + 2ac^4d^2e^4 + 36a^3cd^3e^4 + 44a^2bcd^3e^4 + 12ab^2cd^3e^4 + 32a^2c^2d^3e^4 + 16abc^2d^3e^4 + 4ac^3d^3e^4 + 2a^2cd^4e^4 + 2abcd^4e^4 + 2ac^2d^4e^4 + 46a^4cde^5 + 77a^3bcde^5 + 43a^2b^2cde^5 + 8ab^3cde^5 + 68a^3c^2de^5 + 79a^2bc^2de^5 + 24ab^2c^2de^5 + 32a^2c^3de^5 + 20abc^3de^5 + 4ac^4de^5 + 50a^3cd^2e^5 + 62a^2bcd^2e^5 + 20ab^2cd^2e^5 + 52a^2c^2d^2e^5 + 32abc^2d^2e^5 + 12ac^3d^2e^5 + 8a^2cd^3e^5 + 8abcd^3e^5 + 8ac^2d^3e^5 + 22a^3cde^6 + 28a^2bcde^6 + 10ab^2cde^6 + 26a^2c^2de^6 + 18abc^2de^6 + 8ac^3de^6 + 10a^2cd^2e^6 + 10abcd^2e^6 + 10ac^2d^2e^6 + 4a^2cde^7 + 4abcde^7 + 4ac^2de^7$

$$\rho_6^G(a,b,c,d,e) =$$

$4a^6bcd^2e + 8a^5b^2cd^2e + 5a^4b^3cd^2e + a^3b^4cd^2e + 8a^5bc^2d^2e + 14a^4b^2c^2d^2e + 7a^3b^3c^2d^2e + a^2b^4c^2d^2e + 5a^4bc^3d^2e + 7a^3b^2c^3d^2e + 2a^2b^3c^3d^2e + a^3bc^4d^2e + a^2b^2c^4d^2e + 10a^5bcd^3e + 15a^4b^2cd^3e + 7a^3b^3cd^3e + a^2b^4cd^3e + 15a^4bc^2d^3e + 16a^3b^2c^2d^3e + 4a^2b^3c^2d^3e + 7a^3bc^3d^3e + 4a^2b^2c^3d^3e + a^2bc^4d^3e + 8a^4bcd^4e + 8a^3b^2cd^4e + 2a^2b^3cd^4e + 8a^3bc^2d^4e + 4a^2b^2c^2d^4e + 2a^2bc^3d^4e + 2a^3bcd^5e + a^2b^2cd^5e + a^2bc^2d^5e + 4a^6bcde^2 + 12a^5b^2cde^2 + 13a^4b^3cde^2 + 6a^3b^4cde^2 + a^2b^5cde^2 + 12a^5bc^2de^2 + 34a^4b^2c^2de^2 + 34a^3b^3c^2de^2 + 14a^2b^4c^2de^2 + 2ab^5c^2de^2 + 9a^4bc^3de^2 + 22a^3b^2c^3de^2 + 17a^2b^3c^3de^2 + 4ab^4c^3de^2 + 2a^3bc^4de^2 + 4a^2b^2c^4de^2 + 2ab^3c^4de^2 + 28a^5bcd^2e^2 + 58a^4b^2cd^2e^2 + 44a^3b^3cd^2e^2 + 15a^2b^4cd^2e^2 + 2ab^5cd^2e^2 + 48a^4bc^2d^2e^2 + 83a^3b^2c^2d^2e^2 + 47a^2b^3c^2d^2e^2 + 9ab^4c^2d^2e^2 + 25a^3bc^3d^2e^2 + 32a^2b^2c^3d^2e^2 + 10ab^3c^3d^2e^2 + 4a^2bc^4d^2e^2 + 3ab^2c^4d^2e^2 + 40a^4bcd^3e^2 + 58a^3b^2cd^3e^2 + 29a^2b^3cd^3e^2 + 5ab^4cd^3e^2 + 46a^3bc^2d^3e^2 + 46a^2b^2c^2d^3e^2 + 12ab^3c^2d^3e^2 + 15a^2bc^3d^3e^2 + 8ab^2c^3d^3e^2 + abc^4d^3e^2 + 18a^3bcd^4e^2 + 17a^2b^2cd^4e^2 + 4ab^3cd^4e^2 + 13a^2bc^2d^4e^2 + 6ab^2c^2d^4e^2 + 2abc^3d^4e^2 + 2a^2bcd^5e^2 + ab^2cd^5e^2 + abc^2d^5e^2 + 20a^5bcde^3 + 50a^4b^2cde^3 + 44a^3b^3cde^3 + 16a^2b^4cde^3 + 2ab^5cde^3 + 44a^4bc^2de^3 + 91a^3b^2c^2de^3 + 59a^2b^3c^2de^3 + 12ab^4c^2de^3 + 29a^3bc^3de^3 + 45a^2b^2c^3de^3 + 16ab^3c^3de^3 + 6a^2bc^4de^3 + 6ab^2c^4de^3 + 65a^4bcd^2e^3 + 113a^3b^2cd^2e^3 + 66a^2b^3cd^2e^3 + 13ab^4cd^2e^3 + 87a^3bc^2d^2e^3 + 109a^2b^2c^2d^2e^3 + 34ab^3c^2d^2e^3 + 34a^2bc^3d^2e^3 + 24ab^2c^3d^2e^3 + 3abc^4d^2e^3 + 51a^3bcd^3e^3 + 57a^2b^2cd^3e^3 + 16ab^3cd^3e^3 + 42a^2bc^2d^3e^3 + 24ab^2c^2d^3e^3 + 8abc^3d^3e^3 + 10a^2bcd^4e^3 + 5ab^2cd^4e^3 + 5abc^2d^4e^3 + 36a^4bcde^4 + 68a^3b^2cde^4 + 41a^2b^3cde^4 + 8ab^4cde^4 + 58a^3bc^2de^4 + 77a^2b^2c^2de^4 + 24ab^3c^2de^4 + 28a^2bc^3de^4 + 20ab^2c^3de^4 + 4abc^4de^4 + 62a^3bcd^2e^4 + 74a^2b^2cd^2e^4 + 22ab^3cd^2e^4 + 58a^2bc^2d^2e^4 + 36ab^2c^2d^2e^4 + 14abc^3d^2e^4 + 21a^2bcd^3e^4 + 11ab^2cd^3e^4 + 11abc^2d^3e^4 + 28a^3bcde^5 + 34a^2b^2cde^5 + 10ab^3cde^5 + 30a^2bc^2de^5 + 18ab^2c^2de^5 + 8abc^3de^5 + 21a^2bcd^2e^5 + 11ab^2cd^2e^5 + 11abc^2d^2e^5 + 8a^2bcde^6 + 4ab^2cde^6 + 4abc^2de^6$

$$\rho_7^G(a,b,c,d,e) =$$

$4a^6bcd^3 + 8a^5b^2cd^3 + 5a^4b^3cd^3 + a^3b^4cd^3 + 8a^5bc^2d^3 + 10a^4b^2c^2d^3 + 3a^3b^3c^2d^3 + 5a^4bc^3d^3 + 3a^3b^2c^3d^3 + a^3bc^4d^3 + 8a^5bcd^4 + 10a^4b^2cd^4 + 3a^3b^3cd^4 + 10a^4bc^2d^4 + 6a^3b^2c^2d^4 + 3a^3bc^3d^4 + 4a^4bcd^5 + 2a^3b^2cd^5 + 2a^3bc^2d^5 + 4a^6bcd^2e + 12a^5b^2cd^2e + 13a^4b^3cd^2e + 6a^3b^4cd^2e + a^2b^5cd^2e + 8a^5bc^2d^2e + 18a^4b^2c^2d^2e + 13a^3b^3c^2d^2e + 3a^2b^4c^2d^2e + 5a^4bc^3d^2e + 8a^3b^2c^3d^2e + 3a^2b^3c^3d^2e + a^3bc^4d^2e + a^2b^2c^4d^2e + 22a^5bcd^3e + 39a^4b^2cd^3e + 22a^3b^3cd^3e + 4a^2b^4cd^3e + 33a^4bc^2d^3e + 37a^3b^2c^2d^3e + 10a^2b^3c^2d^3e + 15a^3bc^3d^3e + 8a^2b^2c^3d^3e + 2a^2bc^4d^3e + 24a^4bcd^4e + 24a^3b^2cd^4e + 6a^2b^3cd^4e + 22a^3bc^2d^4e + 11a^2b^2c^2d^4e + 5a^2bc^3d^4e + 6a^3bcd^5e + 3a^2b^2cd^5e + 3a^2bc^2d^5e + 16a^5bcd^2e^2 + 36a^4b^2cd^2e^2 + 26a^3b^3cd^2e^2 + 6a^2b^4cd^2e^2 + 26a^4bc^2d^2e^2 + 41a^3b^2c^2d^2e^2 + 17a^2b^3c^2d^2e^2 + ab^4c^2d^2e^2 + 13a^3bc^3d^2e^2 + 13a^2b^2c^3d^2e^2 + 2ab^3c^3d^2e^2 +$

$2a^2bc^4d^2e^2 + ab^2c^4d^2e^2 + 40a^4bcd^3e^2 + 52a^3b^2cd^3e^2 + 18a^2b^3cd^3e^2 + ab^4cd^3e^2 + 44a^3bc^2d^3e^2 +$
$34a^2b^2c^2d^3e^2 + 4ab^3c^2d^3e^2 + 14a^2bc^3d^3e^2 + 4ab^2c^3d^3e^2 + abc^4d^3e^2 + 22a^3bcd^4e^2 + 15a^2b^2cd^4e^2 +$
$2ab^3cd^4e^2 + 15a^2bc^2d^4e^2 + 4ab^2c^2d^4e^2 + 2abc^3d^4e^2 + 2a^2bcd^5e^2 + ab^2cd^5e^2 + abc^2d^5e^2 + 23a^4bcd^2e^3 +$
$35a^3b^2cd^2e^3 + 14a^2b^3cd^2e^3 + ab^4cd^2e^3 + 29a^3bc^2d^2e^3 + 27a^2b^2c^2d^2e^3 + 4ab^3c^2d^2e^3 + 10a^2bc^3d^2e^3 +$
$4ab^2c^3d^2e^3 + abc^4d^2e^3 + 29a^3bcd^3e^3 + 23a^2b^2cd^3e^3 + 4ab^3cd^3e^3 + 22a^2bc^2d^3e^3 + 8ab^2c^2d^3e^3 +$
$4abc^3d^3e^3 + 6a^2bcd^4e^3 + 3ab^2cd^4e^3 + 3abc^2d^4e^3 + 14a^3bcd^2e^4 + 12a^2b^2cd^2e^4 + 2ab^3cd^2e^4 + 12a^2bc^2d^2e^4 +$
$4ab^2c^2d^2e^4 + 2abc^3d^2e^4 + 7a^2bcd^3e^4 + 3ab^2cd^3e^4 + 3abc^2d^3e^4 + 3a^2bcd^2e^5 + ab^2cd^2e^5 + abc^2d^2e^5$

$$\rho_8^G(a, b, c, d, e) =$$

$4a^7cd^3 + 8a^6bcd^3 + 5a^5b^2cd^3 + a^4b^3cd^3 + 8a^6c^2d^3 + 10a^5bc^2d^3 + 3a^4b^2c^2d^3 + 5a^5c^3d^3 + 3a^4bc^3d^3 +$
$a^4c^4d^3 + 8a^6cd^4 + 10a^5bcd^4 + 3a^4b^2cd^4 + 10a^5c^2d^4 + 6a^4bc^2d^4 + 3a^4c^3d^4 + 4a^5cd^5 + 2a^4bcd^5 + 2a^4c^2d^5 +$
$4a^7cd^2e + 12a^6bcd^2e + 13a^5b^2cd^2e + 6a^4b^3cd^2e + a^3b^4cd^2e + 8a^6c^2d^2e + 18a^5bc^2d^2e + 13a^4b^2c^2d^2e +$
$3a^3b^3c^2d^2e + 5a^5c^3d^2e + 8a^4bc^3d^2e + 3a^3b^2c^3d^2e + a^4c^4d^2e + a^3bc^4d^2e + 26a^6cd^3e + 47a^5bcd^3e +$
$27a^4b^2cd^3e + 5a^3b^3cd^3e + 43a^5c^2d^3e + 50a^4bc^2d^3e + 14a^3b^2c^2d^3e + 23a^4c^3d^3e + 13a^3bc^3d^3e +$
$4a^3c^4d^3e + 32a^5cd^4e + 35a^4bcd^4e + 10a^3b^2cd^4e + 35a^4c^2d^4e + 20a^3bc^2d^4e + 10a^3c^3d^4e + 10a^4cd^5e +$
$6a^3bcd^5e + 6a^3c^2d^5e + 18a^6cd^2e^2 + 41a^5bcd^2e^2 + 30a^4b^2cd^2e^2 + 7a^3b^3cd^2e^2 + 33a^5c^2d^2e^2 + 56a^4bc^2d^2e^2 +$
$27a^3b^2c^2d^2e^2 + 3a^2b^3c^2d^2e^2 + 20a^4c^3d^2e^2 + 24a^3bc^3d^2e^2 + 6a^2b^2c^3d^2e^2 + 4a^3c^4d^2e^2 + 3a^2bc^4d^2e^2 +$
$60a^5cd^3e^2 + 86a^4bcd^3e^2 + 36a^3b^2cd^3e^2 + 4a^2b^3cd^3e^2 + 85a^4c^2d^3e^2 + 79a^3bc^2d^3e^2 + 16a^2b^2c^2d^3e^2 +$
$38a^3c^3d^3e^2 + 17a^2bc^3d^3e^2 + 5a^2c^4d^3e^2 + 46a^4cd^4e^2 + 42a^3bcd^4e^2 + 10a^2b^2cd^4e^2 + 45a^3c^2d^4e^2 +$
$21a^2bc^2d^4e^2 + 11a^2c^3d^4e^2 + 8a^3cd^5e^2 + 6a^2bcd^5e^2 + 6a^2c^2d^5e^2 + 32a^5cd^2e^3 + 54a^4bcd^2e^3 + 27a^3b^2cd^2e^3 +$
$4a^2b^3cd^2e^3 + 52a^4c^2d^2e^3 + 63a^3bc^2d^2e^3 + 20a^2b^2c^2d^2e^3 + 2ab^3c^2d^2e^3 + 28a^3c^3d^2e^3 + 23a^2bc^3d^2e^3 +$
$4ab^2c^3d^2e^3 + 5a^2c^4d^2e^3 + 2abc^4d^2e^3 + 64a^4cd^3e^3 + 71a^3bcd^3e^3 + 24a^2b^2cd^3e^3 + 2ab^3cd^3e^3 + 78a^3c^2d^3e^3 +$
$54a^2bc^2d^3e^3 + 8ab^2c^2d^3e^3 + 28a^2c^3d^3e^3 + 8abc^3d^3e^3 + 2ac^4d^3e^3 + 28a^3cd^4e^3 + 24a^2bcd^4e^3 + 4ab^2cd^4e^3 +$
$26a^2c^2d^4e^3 + 8abc^2d^4e^3 + 4ac^3d^4e^3 + 2a^2cd^5e^3 + 2abcd^5e^3 + 2ac^2d^5e^3 + 28a^4cd^2e^4 + 35a^3bcd^2e^4 +$
$14a^2b^2cd^2e^4 + 2ab^3cd^2e^4 + 39a^3c^2d^2e^4 + 33a^2bc^2d^2e^4 + 8ab^2c^2d^2e^4 + 17a^2c^3d^2e^4 + 8abc^3d^2e^4 +$
$2ac^4d^2e^4 + 32a^3cd^3e^4 + 30a^2bcd^3e^4 + 8ab^2cd^3e^4 + 34a^2c^2d^3e^4 + 16abc^2d^3e^4 + 8ac^3d^3e^4 + 6a^2cd^4e^4 +$
$6abcd^4e^4 + 6ac^2d^4e^4 + 12a^3cd^2e^5 + 12a^2bcd^2e^5 + 4ab^2cd^2e^5 + 14a^2c^2d^2e^5 + 8abc^2d^2e^5 + 4ac^3d^2e^5 +$
$6a^2cd^3e^5 + 6abcd^3e^5 + 6ac^2d^3e^5 + 2a^2cd^2e^6 + 2abcd^2e^6 + 2ac^2d^2e^6$

$$\rho_9^G(a, b, c, d, e) =$$

$4a^7cd^2e + 12a^6bcd^2e + 13a^5b^2cd^2e + 6a^4b^3cd^2e + a^3b^4cd^2e + 8a^6c^2d^2e + 18a^5bc^2d^2e + 13a^4b^2c^2d^2e +$
$3a^3b^3c^2d^2e + 5a^5c^3d^2e + 8a^4bc^3d^2e + 3a^3b^2c^3d^2e + a^4c^4d^2e + a^3bc^4d^2e + 8a^6cd^3e + 18a^5bcd^3e +$
$13a^4b^2cd^3e + 3a^3b^3cd^3e + 10a^5c^2d^3e + 16a^4bc^2d^3e + 6a^3b^2c^2d^3e + 3a^4c^3d^3e + 3a^3bc^3d^3e + 4a^5cd^4e +$
$6a^4bcd^4e + 2a^3b^2cd^4e + 2a^4c^2d^4e + 2a^3bc^2d^4e + 18a^6cd^2e^2 + 39a^5bcd^2e^2 + 27a^4b^2cd^2e^2 + 6a^3b^3cd^2e^2 +$
$33a^5c^2d^2e^2 + 49a^4bc^2d^2e^2 + 19a^3b^2c^2d^2e^2 + a^2b^3c^2d^2e^2 + 20a^4c^3d^2e^2 + 17a^3bc^3d^2e^2 + 2a^2b^2c^3d^2e^2 +$
$4a^3c^4d^2e^2 + a^2bc^4d^2e^2 + 28a^5cd^3e^2 + 41a^4bcd^3e^2 + 16a^3b^2cd^3e^2 + a^2b^3cd^3e^2 + 33a^4c^2d^3e^2 + 28a^3bc^2d^3e^2 +$
$3a^2b^2c^2d^3e^2 + 10a^3c^3d^3e^2 + 2a^2bc^3d^3e^2 + 10a^4cd^4e^2 + 8a^3bcd^4e^2 + a^2b^2cd^4e^2 + 6a^3c^2d^4e^2 + a^2bc^2d^4e^2 +$
$32a^5cd^2e^3 + 47a^4bcd^2e^3 + 18a^3b^2cd^2e^3 + a^2b^3cd^2e^3 + 52a^4c^2d^2e^3 + 47a^3bc^2d^2e^3 + 8a^2b^2c^2d^2e^3 +$
$28a^3c^3d^2e^3 + 12a^2bc^3d^2e^3 + 5a^2c^4d^2e^3 + 36a^4cd^3e^3 + 34a^3bcd^3e^3 + 8a^2b^2cd^3e^3 + 39a^3c^2d^3e^3 +$
$19a^2bc^2d^3e^3 + 11a^2c^3d^3e^3 + 8a^3cd^4e^3 + 6a^2bcd^4e^3 + 6a^2c^2d^4e^3 + 28a^4cd^2e^4 + 28a^3bcd^2e^4 + 7a^2b^2cd^2e^4 +$
$39a^3c^2d^2e^4 + 22a^2bc^2d^2e^4 + 2ab^2c^2d^2e^4 + 17a^2c^3d^2e^4 + 4abc^3d^2e^4 + 2ac^4d^2e^4 + 20a^3cd^3e^4 + 16a^2bcd^3e^4 +$
$2ab^2cd^3e^4 + 20a^2c^2d^3e^4 + 6abc^2d^3e^4 + 4ac^3d^3e^4 + 2a^2cd^4e^4 + 2abcd^4e^4 + 2ac^2d^4e^4 + 12a^3cd^2e^5 +$

$10a^2bcd^2e^5 + 2ab^2cd^2e^5 + 14a^2c^2d^2e^5 + 6abc^2d^2e^5 + 4ac^3d^2e^5 + 4a^2cd^3e^5 + 4abcd^3e^5 + 4ac^2d^3e^5 + 2a^2cd^2e^6 + 2abcd^2e^6 + 2ac^2d^2e^6$

$$\rho_{10}^G(a,b,c,d,e) =$$

$8a^7cde^2 + 28a^6bcde^2 + 38a^5b^2cde^2 + 25a^4b^3cde^2 + 8a^3b^4cde^2 + a^2b^5cde^2 + 16a^6c^2de^2 + 48a^5bc^2de^2 + 52a^4b^2c^2de^2 + 24a^3b^3c^2de^2 + 4a^2b^4c^2de^2 + 10a^5c^3de^2 + 25a^4bc^3de^2 + 20a^3b^2c^3de^2 + 5a^2b^3c^3de^2 + 2a^4c^4de^2 + 4a^3bc^4de^2 + 2a^2b^2c^4de^2 + 16a^6cd^2e^2 + 52a^5bcd^2e^2 + 60a^4b^2cd^2e^2 + 29a^3b^3cd^2e^2 + 5a^2b^4cd^2e^2 + 20a^5c^2d^2e^2 + 60a^4bc^2d^2e^2 + 51a^3b^2c^2d^2e^2 + 13a^2b^3c^2d^2e^2 + 6a^4c^3d^2e^2 + 20a^3bc^3d^2e^2 + 10a^2b^2c^3d^2e^2 + 2a^2bc^4d^2e^2 + 8a^5cd^3e^2 + 30a^4bcd^3e^2 + 27a^3b^2cd^3e^2 + 7a^2b^3cd^3e^2 + 4a^4c^2d^3e^2 + 23a^3bc^2d^3e^2 + 12a^2b^2c^2d^3e^2 + 5a^2bc^3d^3e^2 + 6a^3bcd^4e^2 + 3a^2b^2cd^4e^2 + 3a^2bc^2d^4e^2 + 4a^7ce^3 + 16a^6bce^3 + 25a^5b^2ce^3 + 19a^4b^3ce^3 + 7a^3b^4ce^3 + a^2b^5ce^3 + 12a^6c^2e^3 + 46a^5bc^2e^3 + 68a^4b^2c^2e^3 + 48a^3b^3c^2e^3 + 16a^2b^4c^2e^3 + 2ab^5c^2e^3 + 9a^5c^3e^3 + 31a^4bc^3e^3 + 39a^3b^2c^3e^3 + 21a^2b^3c^3e^3 + 4ab^4c^3e^3 + 2a^4c^4e^3 + 6a^3bc^4e^3 + 6a^2b^2c^4e^3 + 2ab^3c^4e^3 + 48a^6cde^3 + 150a^5bcde^3 + 177a^4b^2cde^3 + 97a^3b^3cde^3 + 24a^2b^4cde^3 + 2ab^5cde^3 + 84a^5c^2de^3 + 221a^4bc^2de^3 + 205a^3b^2c^2de^3 + 78a^2b^3c^2de^3 + 10ab^4c^2de^3 + 46a^4c^3de^3 + 96a^3bc^3de^3 + 62a^2b^2c^3de^3 + 12ab^3c^3de^3 + 8a^3c^4de^3 + 12a^2bc^4de^3 + 4ab^2c^4de^3 + 64a^5cd^2e^3 + 178a^4bcd^2e^3 + 163a^3b^2cd^2e^3 + 57a^2b^3cd^2e^3 + 6ab^4cd^2e^3 + 70a^4c^2d^2e^3 + 167a^3bc^2d^2e^3 + 102a^2b^2c^2d^2e^3 + 16ab^3c^2d^2e^3 + 20a^3c^3d^2e^3 + 41a^2bc^3d^2e^3 + 12ab^2c^3d^2e^3 + 2abc^4d^2e^3 + 20a^4cd^3e^3 + 62a^3bcd^3e^3 + 38a^2b^2cd^3e^3 + 6ab^3cd^3e^3 + 12a^3c^2d^3e^3 + 34a^2bc^2d^3e^3 + 10ab^2c^2d^3e^3 + 4abc^3d^3e^3 + 4a^2bcd^4e^3 + 2ab^2cd^4e^3 + 2abc^2d^4e^3 + 22a^6ce^4 + 77a^5bce^4 + 103a^4b^2ce^4 + 65a^3b^3ce^4 + 19a^2b^4ce^4 + 2ab^5ce^4 + 51a^5c^2e^4 + 157a^4bc^2e^4 + 175a^3b^2c^2e^4 + 83a^2b^3c^2e^4 + 14ab^4c^2e^4 + 36a^4c^3e^4 + 92a^3bc^3e^4 + 76a^2b^2c^3e^4 + 20ab^3c^3e^4 + 8a^3c^4e^4 + 16a^2bc^4e^4 + 8ab^2c^4e^4 + 110a^5cde^4 + 294a^4bcde^4 + 281a^3b^2cde^4 + 113a^2b^3cde^4 + 16ab^4cde^4 + 164a^4c^2de^4 + 345a^3bc^2de^4 + 230a^2b^2c^2de^4 + 48ab^3c^2de^4 + 76a^3c^3de^4 + 113a^2bc^3de^4 + 40ab^2c^3de^4 + 10a^2c^4de^4 + 8abc^4de^4 + 92a^4cd^2e^4 + 207a^3bcd^2e^4 + 134a^2b^2cd^2e^4 + 26ab^3cd^2e^4 + 90a^3c^2d^2e^4 + 144a^2bc^2d^2e^4 + 46ab^2c^2d^2e^4 + 22a^2c^3d^2e^4 + 20abc^3d^2e^4 + 16a^3cd^3e^4 + 34a^2bcd^3e^4 + 12ab^2cd^3e^4 + 12a^2c^2d^3e^4 + 12abc^2d^3e^4 + 46a^5ce^5 + 134a^4bce^5 + 141a^3b^2ce^5 + 63a^2b^3ce^5 + 10ab^4ce^5 + 85a^4c^2e^5 + 199a^3bc^2e^5 + 150a^2b^2c^2e^5 + 36ab^3c^2e^5 + 51a^3c^3e^5 + 87a^2bc^3e^5 + 36ab^2c^3e^5 + 10a^2c^4e^5 + 10abc^4e^5 + 120a^4cde^5 + 258a^3bcde^5 + 176a^2b^2cde^5 + 38ab^3cde^5 + 150a^3c^2de^5 + 222a^2bc^2de^5 + 78ab^2c^2de^5 + 56a^2c^3de^5 + 44abc^3de^5 + 4ac^4de^5 + 56a^3cd^2e^5 + 92a^2bcd^2e^5 + 32ab^2cd^2e^5 + 52a^2c^2d^2e^5 + 40abc^2d^2e^5 + 8ac^3d^2e^5 + 4a^2cd^3e^5 + 4abcd^3e^5 + 4ac^2d^3e^5 + 46a^4ce^6 + 105a^3bce^6 + 77a^2b^2ce^6 + 18ab^3ce^6 + 68a^3c^2e^6 + 109a^2bc^2e^6 + 42ab^2c^2e^6 + 32a^2c^3e^6 + 28abc^3e^6 + 4ac^4e^6 + 62a^3cde^6 + 98a^2bcde^6 + 36ab^2cde^6 + 66a^2c^2de^6 + 52abc^2de^6 + 16ac^3de^6 + 12a^2cd^2e^6 + 12abcd^2e^6 + 12ac^2d^2e^6 + 22a^3ce^7 + 36a^2bce^7 + 14ab^2ce^7 + 26a^2c^2e^7 + 22abc^2e^7 + 8ac^3e^7 + 12a^2cde^7 + 12abcde^7 + 12ac^2de^7 + 4a^2ce^8 + 4abce^8 + 4ac^2e^8$

$$\rho_{11}^G(a,b,c,d,e) =$$

$8a^6bcde^2 + 28a^5b^2cde^2 + 38a^4b^3cde^2 + 25a^3b^4cde^2 + 8a^2b^5cde^2 + ab^6cde^2 + 16a^5bc^2de^2 + 48a^4b^2c^2de^2 + 52a^3b^3c^2de^2 + 24a^2b^4c^2de^2 + 4ab^5c^2de^2 + 10a^4bc^3de^2 + 25a^3b^2c^3de^2 + 20a^2b^3c^3de^2 + 5ab^4c^3de^2 + 2a^3bc^4de^2 + 4a^2b^2c^4de^2 + 2ab^3c^4de^2 + 24a^5bcd^2e^2 + 72a^4b^2cd^2e^2 + 78a^3b^3cd^2e^2 + 36a^2b^4cd^2e^2 + 6ab^5cd^2e^2 + 36a^4bc^2d^2e^2 + 92a^3b^2c^2d^2e^2 + 71a^2b^3c^2d^2e^2 + 17ab^4c^2d^2e^2 + 16a^3bc^3d^2e^2 + 35a^2b^2c^3d^2e^2 + 15ab^3c^3d^2e^2 + 2a^2bc^4d^2e^2 + 4ab^2c^4d^2e^2 + 30a^4bcd^3e^2 + 71a^3b^2cd^3e^2 + 52a^2b^3cd^3e^2 + 12ab^4cd^3e^2 + 37a^3bc^2d^3e^2 + 65a^2b^2c^2d^3e^2 + 25ab^3c^2d^3e^2 + 15a^2bc^3d^3e^2 + 15ab^2c^3d^3e^2 + 2abc^4d^3e^2 + 20a^3bcd^4e^2 + 30a^2b^2cd^4e^2 + 10ab^3cd^4e^2 + 20a^2bc^2d^4e^2 + 15ab^2c^2d^4e^2 + 5abc^3d^4e^2 + 6a^2bcd^5e^2 + 3ab^2cd^5e^2 + 3abc^2d^5e^2 + 4a^6bce^3 + 16a^5b^2ce^3 + 25a^4b^3ce^3 + 19a^3b^4ce^3 + 7a^2b^5ce^3 + ab^6ce^3 + 12a^5bc^2e^3 + 46a^4b^2c^2e^3 + 68a^3b^3c^2e^3 + 48a^2b^4c^2e^3 + 16ab^5c^2e^3 + 2b^6c^2e^3 + 9a^4bc^3e^3 + 31a^3b^2c^3e^3 + 39a^2b^3c^3e^3 +$

$$21ab^4c^3e^3 + 4b^5c^3e^3 + 2a^3bc^4e^3 + 6a^2b^2c^4e^3 + 6ab^3c^4e^3 + 2b^4c^4e^3 + 52a^5bcde^3 + 162a^4b^2cde^3 +$$
$$190a^3b^3cde^3 + 103a^2b^4cde^3 + 25ab^5cde^3 + 2b^6cde^3 + 96a^4bc^2de^3 + 255a^3b^2c^2de^3 + 239a^2b^3c^2de^3 +$$
$$92ab^4c^2de^3 + 12b^5c^2de^3 + 55a^3bc^3de^3 + 118a^2b^2c^3de^3 + 79ab^3c^3de^3 + 16b^4c^3de^3 + 10a^2bc^4de^3 +$$
$$16ab^2c^4de^3 + 6b^3c^4de^3 + 110a^4bcd^2e^3 + 275a^3b^2cd^2e^3 + 234a^2b^3cd^2e^3 + 78ab^4cd^2e^3 + 8b^5cd^2e^3 +$$
$$151a^3bc^2d^2e^3 + 299a^2b^2c^2d^2e^3 + 168ab^3c^2d^2e^3 + 26b^4c^2d^2e^3 + 65a^2bc^3d^2e^3 + 90ab^2c^3d^2e^3 + 24b^3c^3d^2e^3 +$$
$$8abc^4d^2e^3 + 6b^2c^4d^2e^3 + 98a^3bcd^3e^3 + 174a^2b^2cd^3e^3 + 87ab^3cd^3e^3 + 12b^4cd^3e^3 + 106a^2bc^2d^3e^3 +$$
$$119ab^2c^2d^3e^3 + 26b^3c^2d^3e^3 + 32abc^3d^3e^3 + 16b^2c^3d^3e^3 + 2bc^4d^3e^3 + 40a^2bcd^4e^3 + 37ab^2cd^4e^3 +$$
$$8b^3cd^4e^3 + 29abc^2d^4e^3 + 12b^2c^2d^4e^3 + 4bc^3d^4e^3 + 4abcd^5e^3 + 2b^2cd^5e^3 + 2bc^2d^5e^3 + 22a^5bce^4 +$$
$$77a^4b^2ce^4 + 103a^3b^3ce^4 + 65a^2b^4ce^4 + 19ab^5ce^4 + 2b^6ce^4 + 51a^4bc^2e^4 + 157a^3b^2c^2e^4 + 175a^2b^3c^2e^4 +$$
$$83ab^4c^2e^4 + 14b^5c^2e^4 + 36a^3bc^3e^4 + 92a^2b^2c^3e^4 + 76ab^3c^3e^4 + 20b^4c^3e^4 + 8a^2bc^4e^4 + 16ab^2c^4e^4 +$$
$$8b^3c^4e^4 + 130a^4bcde^4 + 344a^3b^2cde^4 + 325a^2b^3cde^4 + 129ab^4cde^4 + 18b^5cde^4 + 208a^3bc^2de^4 +$$
$$436a^2b^2c^2de^4 + 289ab^3c^2de^4 + 60b^4c^2de^4 + 105a^2bc^3de^4 + 158ab^2c^3de^4 + 56b^3c^3de^4 + 16abc^4de^4 +$$
$$14b^2c^4de^4 + 187a^3bcd^2e^4 + 364a^2b^2cd^2e^4 + 217ab^3cd^2e^4 + 40b^4cd^2e^4 + 222a^2bc^2d^2e^4 + 292ab^2c^2d^2e^4 +$$
$$86b^3c^2d^2e^4 + 77abc^3d^2e^4 + 52b^2c^3d^2e^4 + 6bc^4d^2e^4 + 105a^2bcd^3e^4 + 123ab^2cd^3e^4 + 34b^3cd^3e^4 +$$
$$89abc^2d^3e^4 + 50b^2c^2d^3e^4 + 16bc^3d^3e^4 + 18abcd^4e^4 + 10b^2cd^4e^4 + 10bc^2d^4e^4 + 46a^4bce^5 + 134a^3b^2ce^5 +$$
$$141a^2b^3ce^5 + 63ab^4ce^5 + 10b^5ce^5 + 85a^3bc^2e^5 + 199a^2b^2c^2e^5 + 150ab^3c^2e^5 + 36b^4c^2e^5 + 51a^2bc^3e^5 +$$
$$87ab^2c^3e^5 + 36b^3c^3e^5 + 10abc^4e^5 + 10b^2c^4e^5 + 156a^3bcde^5 + 326a^2b^2cde^5 + 217ab^3cde^5 + 46b^4cde^5 +$$
$$208a^2bc^2de^5 + 299ab^2c^2de^5 + 102b^3c^2de^5 + 84abc^3de^5 + 64b^2c^3de^5 + 8bc^4de^5 + 139a^2bcd^2e^5 +$$
$$185ab^2cd^2e^5 + 58b^3cd^2e^5 + 133abc^2d^2e^5 + 86b^2c^2d^2e^5 + 28bc^3d^2e^5 + 36abcd^3e^5 + 22b^2cd^3e^5 +$$
$$22bc^2d^3e^5 + 46a^3bce^6 + 105a^2b^2ce^6 + 77ab^3ce^6 + 18b^4ce^6 + 68a^2bc^2e^6 + 109ab^2c^2e^6 + 42b^3c^2e^6 +$$
$$32abc^3e^6 + 28b^2c^3e^6 + 4bc^4e^6 + 90a^2bcde^6 + 132ab^2cde^6 + 46b^3cde^6 + 96abc^2de^6 + 70b^2c^2de^6 +$$
$$24bc^3de^6 + 38abcd^2e^6 + 26b^2cd^2e^6 + 26bc^2d^2e^6 + 22a^2bce^7 + 36ab^2ce^7 + 14b^3ce^7 + 26abc^2e^7 +$$
$$22b^2c^2e^7 + 8bc^3e^7 + 20abcde^7 + 16b^2cde^7 + 16bc^2de^7 + 4abce^8 + 4b^2ce^8 + 4bc^2e^8$$

$$\rho_{12}^{G}(a,b,c,d,e) =$$

$$4a^6bcd^2e + 12a^5b^2cd^2e + 13a^4b^3cd^2e + 6a^3b^4cd^2e + a^2b^5cd^2e + 8a^5bc^2d^2e + 18a^4b^2c^2d^2e + 13a^3b^3c^2d^2e +$$
$$3a^2b^4c^2d^2e + 5a^4bc^3d^2e + 8a^3b^2c^3d^2e + 3a^2b^3c^3d^2e + a^3bc^4d^2e + a^2b^2c^4d^2e + 12a^5bcd^3e + 26a^4b^2cd^3e +$$
$$18a^3b^3cd^3e + 4a^2b^4cd^3e + 18a^4bc^2d^3e + 26a^3b^2c^2d^3e + 9a^2b^3c^2d^3e + 8a^3bc^3d^3e + 6a^2b^2c^3d^3e +$$
$$a^2bc^4d^3e + 12a^4bcd^4e + 16a^3b^2cd^4e + 5a^2b^3cd^4e + 12a^3bc^2d^4e + 8a^2b^2c^2d^4e + 3a^2bc^3d^4e + 4a^3bcd^5e +$$
$$2a^2b^2cd^5e + 2a^2bc^2d^5e + 18a^5bcd^2e^2 + 39a^4b^2cd^2e^2 + 27a^3b^3cd^2e^2 + 6a^2b^4cd^2e^2 + 33a^4bc^2d^2e^2 +$$
$$49a^3b^2c^2d^2e^2 + 19a^2b^3c^2d^2e^2 + ab^4c^2d^2e^2 + 20a^3bc^3d^2e^2 + 17a^2b^2c^3d^2e^2 + 2ab^3c^3d^2e^2 + 4a^2bc^4d^2e^2 +$$
$$ab^2c^4d^2e^2 + 38a^4bcd^3e^2 + 54a^3b^2cd^3e^2 + 20a^2b^3cd^3e^2 + ab^4cd^3e^2 + 48a^3bc^2d^3e^2 + 39a^2b^2c^2d^3e^2 +$$
$$4ab^3c^2d^3e^2 + 17a^2bc^3d^3e^2 + 4ab^2c^3d^3e^2 + abc^4d^3e^2 + 22a^3bcd^4e^2 + 16a^2b^2cd^4e^2 + 2ab^3cd^4e^2 +$$
$$16a^2bc^2d^4e^2 + 4ab^2c^2d^4e^2 + 2abc^3d^4e^2 + 2a^2bcd^5e^2 + ab^2cd^5e^2 + abc^2d^5e^2 + 30a^4bcd^2e^3 + 44a^3b^2cd^2e^3 +$$
$$17a^2b^3cd^2e^3 + ab^4cd^2e^3 + 45a^3bc^2d^2e^3 + 39a^2b^2c^2d^2e^3 + 6ab^3c^2d^2e^3 + 21a^2bc^3d^2e^3 + 8ab^2c^3d^2e^3 +$$
$$3abc^4d^2e^3 + 38a^3bcd^3e^3 + 32a^2b^2cd^3e^3 + 6ab^3cd^3e^3 + 35a^2bc^2d^3e^3 + 14ab^2c^2d^3e^3 + 8abc^3d^3e^3 +$$
$$8a^2bcd^4e^3 + 5ab^2cd^4e^3 + 5abc^2d^4e^3 + 21a^3bcd^2e^4 + 19a^2b^2cd^2e^4 + 4ab^3cd^2e^4 + 23a^2bc^2d^2e^4 +$$
$$10ab^2c^2d^2e^4 + 6abc^3d^2e^4 + 11a^2bcd^3e^4 + 7ab^2cd^3e^4 + 7abc^2d^3e^4 + 5a^2bcd^2e^5 + 3ab^2cd^2e^5 + 3abc^2d^2e^5$$

References

[1] H. D. Kim and E. K. O'Shea. A quantitative model of transcription factor-activated gene expression. *Nat. Struct. Mol. Biol.*, 15:1192–8, 2008.

[2] K. M. Ong, J. A. Blackford Jr., B. L. Kagan, S. S. Simons, and C. C. Chow. A theoretical framework for gene induction and experimental comparisons. *Proc. Natl. Acad. Sci. USA*, 107:7107–12, 2010.
